# Influence of Curvature, Growth, and Anisotropy on the Evolution of Turing Patterns on Growing Manifolds

**DOI:** 10.1007/s11538-018-0535-y

**Published:** 2018-12-03

**Authors:** Andrew L. Krause, Meredith A. Ellis, Robert A. Van Gorder

**Affiliations:** Mathematical Institute, University of Oxford, Andrew Wiles Building, Radcliffe Observatory Quarter, Woodstock Road, Oxford, OX2 6GG UK

**Keywords:** Turing patterns, Growing surfaces, Anisotropic growth, Pattern selection, Pattern robustness

## Abstract

We study two-species reaction–diffusion systems on growing manifolds, including situations where the growth is anisotropic yet dilational in nature. In contrast to the literature on linear instabilities in such systems, we study how growth and anisotropy impact the qualitative properties of nonlinear patterned states which have formed before growth is initiated. We produce numerical solutions to numerous reaction–diffusion systems with varying reaction kinetics, manner of growth (both isotropic and anisotropic), and timescales of growth on both planar elliptical and curved ellipsoidal domains. We find that in some parameter regimes, some of these factors have a negligible effect on the long-time patterned state. On the other hand, we find that some of these factors play a role in determining the patterns formed on surfaces and that anisotropic growth can produce qualitatively different patterns to those formed under isotropic growth.

## Introduction

Since Turing first proposed reaction–diffusion systems as a model for pattern formation (Turing 1952), much work has been performed to understand the theoretical and biological aspects of this phenomenon (Satnoianu et al. 2000; Green and Sharpe 2015; Marcon et al. 2016; Woolley et al. 2017). Murray (2003) discusses the importance of domain size and shape on the formation of patterns, and the impact of geometry on the kinds of admissible patterns that can arise due to a Turing instability. Curved and complex geometries have been of recent interest, as these provide for more realistic regimes within which to test morphogenetic hypotheses (Tse et al. 2010; Trinh and Ward 2016; Núñez-López et al. 2017). Since reaction–diffusion systems are more difficult to analyze on growing domains, pattern formation has been considered on different-sized static domains to simulate very slow growth (Varea et al. 1997). This requires the reaction and diffusion of the chemical species to occur on a much faster timescale than the growth and also be independent of the growth. An alternative simplification would be to assume growth occurs on a much faster timescale than reaction and diffusion and consider the quasi-static approximation of the reaction–diffusion system on the final shape of the domain. Since neither of these approximations are likely to be valid for all systems modeling biological growth, we consider reaction–diffusion systems where growth, reaction, and diffusion occur on comparable timescales.

Crampin et al. (1999) explicitly considered uniform and isotropic domain growth in one-dimensional reaction–diffusion systems in the slow and fast growth regimes, demonstrating frequency doubling of the emergent Turing patterns. This approach was discussed in the context of biological patterning problems in Crampin and Maini (2001), Barrass et al. (2006) and extended in Crampin et al. (2002) to consider the influence of non-uniform domain growth on one-dimensional reaction–diffusion systems, including apical or boundary growth. Castillo et al. (2016) considered Turing and Turing–Hopf instabilities in an exponentially and isotropically growing square and suggested that anisotropy and curvature are important considerations for extending their analysis. Following work on characterizing instabilities in non-autonomous reaction–diffusion equations on slowly growing domains in Madzvamuse et al. (2010), Hetzer et al. (2012) derived conditions for diffusion-driven instability on time-dependent manifolds. Plaza et al. (2004) derived a general formulation of reaction–diffusion theory on isotropically evolving one and two-dimensional manifolds, with motivation from biological settings where growth and curvature both play a role in organism development.

Beyond computing linear instability criteria, some authors have analytically explored how patterns change and evolve under growth (Comanici and Golubitsky 2008) by exploiting the framework of amplitude (or Ginzburg–Landau) equations (Cross and Hohenberg 1993; van Saarloos et al. 1994). While analytical results on mode competition and selection can be valuable, these are often extremely limited as they only apply near the bifurcation boundary in the parameter space, and they become computationally intractable in many cases of interest. Additionally, to our knowledge, there does not exist an analytical framework applicable to curved manifolds. In contrast, many more studies have explored such systems numerically, developing a variety of techniques to capture the transient and the final patterned states in reaction–diffusion systems on manifolds (Madzvamuse et al. 2003; Barreira et al. 2011; Macdonald et al. 2013; Madzvamuse and Maini 2007; Tuncer and Madzvamuse 2017). While these numerical methods and their applications will always be isolated to specific instances of reaction–diffusion systems, one can hope to at least explore the kinds of emergent behaviors in candidate systems as a way to understand realistic pattern formation and pattern evolution in more complex settings.

All of the above models of reaction–diffusion systems on growing domains only analyzed the case of isotropic (or apical) growth, which is unable to recapitulate the full range of complex biological structures found in developing organisms (Ubeda-Tomás et al. 2008; Corson et al. 2009; Peaucelle et al. 2015). Investigating arbitrary anisotropic growth in the context of biological patterning is a natural extension to reaction–diffusion theories of pattern formation and has been considered in biomechanical models of growth across a range of tissues and organisms (Menzel 2005; Saez et al. 2007; Bittig et al. 2008; Amar and Jia 2013). Madzvamuse and Barreira (2014) considered anisotropic and concentration-dependent growth in the context of cross-diffusion models, but their emphasis was on cross-diffusion-driven instabilities in complicated settings, rather than on understanding anisotropic growth in simple geometries, which has been suggested as a useful direction (Castillo et al. 2016). Rossi et al. (2016) also studied concentration-dependent growth of a scalar reaction–diffusion equation on a time-dependent manifold. Krause et al. (2018a) investigated the effects of Turing instabilities in two-species reaction–advection–diffusion models on a sphere for several well-known reaction kinetics. They find that advection and the compact geometry of the sphere allow for complex spatiotemporal patterns. Since growth can induce advection-like terms into the morphogen evolution equations (Plaza et al. 2004), it is of interest to consider the effect of anisotropic growth on compact domains, such as the sphere.

Much of the work in reaction–diffusion theory on growing domains has emphasized differences between quasi-static, slow, and fast growth regimes. Growth can lead to a robustness of patterning processes in some scenarios (Crampin et al. 1999), where the period-doubling of pattern splitting from an existing pattern leads to insensitivity to the initial symmetry-breaking perturbation which initiated patterning. Despite such differences, most reaction–diffusion systems give rise to qualitatively similar patterns, with quantities such as the pattern wavelength determined primarily by the final domain size, independent of growth. Determining when different kinds of growth influence the final pattern, and the effects that growth has on patterns, is an important problem to determine the validity of quasi-static approximations. Indeed, other questions arise such as: *To what extent does the rate of deformation, type of growth or extremity of growth affect the patterns formed on the domain, if there is any effect at all?*

In this paper, we generalize the modeling of reaction–diffusion systems on growing manifolds developed by Plaza et al. (2004) to allow for dilational anisotropic growth. The restriction of dilational growth allows for relatively simple governing equations amenable to simulation, yet still allows us to compare between quasi-static, isotropic, and anisotropic growth regimes. We then systematically explore three kinds of reaction kinetics on planar and curved two-dimensional manifolds. We also vary the kinds of growth, both in terms of average growth rate, functional form of the growth, and exploring growth in different stages. To address the questions of when these factors matter, we restrict attention to the evolution of established patterns far from homogeneous equilibrium states that arise due to Turing instabilities. In other words, all parameters are chosen within the Turing space such that these systems will always develop a heterogeneous solution, and we focus on qualitative features which change in such a solution as the manifold grows. This allows us to determine when each of these different growth scenarios gives rise to qualitatively different patterns. We emphasize qualitative differences and very pronounced quantitative effects, but do not focus on small quantitative differences between simulations, as these can depend sensitively on parameters and the initial data. We find that all of these features (growth rates, anisotropy, curvature) can give rise to qualitatively different patterns in some regimes, but that some of them (e.g., growth rate) are typically more important than others (e.g., the functional form of growth), and that some reaction kinetics, and some parameter regimes, are more or less influenced by growth.

The remainder of this paper is organized as follows. In Sect. 2, we develop our models to study two-species reaction–diffusion systems on a general two-dimensional manifold undergoing isotropic or anisotropic, but dilational growth. In Sect. 3, we discuss a systematic framework by which we study the effects of such growth on a range of reaction kinetics, demonstrating systems where patterning is generally robust (e.g., qualitatively independent of growth). In the following sections we shall employ this systematic approach to study Turing patterns on growing domains in the case where the domain is a flat ellipse (Sect. 4) and in the case where the domain is an ellipsoid surface (Sect. 5), the former demonstrating the role of anisotropy and the latter additionally demonstrating the role played by local curvature of the domain. We then discuss the evolution and numerical stability of stripe and target patterns in the presence of growth in Sect. 6, considering both the ellipse and ellipsoid domains. We finally discuss the implications of our results in Sect. 7.

## Mathematical Model for Anisotropic Dilational Growth

We now introduce the model accounting for reaction–diffusion on a growing manifold. We first define the kinds of stationary patterns we are interested in and recall the conditions for diffusion-driven instability. We then introduce the concept of a reaction–diffusion system on a growing manifold and show how to transform this system onto a stationary manifold. This is a generalization of Plaza et al. (2004) where we allow for anisotropic dilational growth. Finally, we apply this general framework to two example manifolds which we will simulate in the following sections: a planar ellipse and the surface of an ellipsoid.

### Turing Instability

We consider a general two-species reaction–diffusion system on a stationary manifold $\varOmega$ given by

1a $$\begin{aligned} \frac{\partial u}{\partial t}&= \delta _1 \nabla ^2 u + f(u,v), \end{aligned}$$

1b $$\begin{aligned} \frac{\partial v}{\partial t}&= \delta _2 \nabla ^2 v + g(u,v), \end{aligned}$$

where *u* and *v* are the two interacting chemical species, $\delta _1$ and $\delta _2$ are the diffusion coefficients of *u* and *v*, respectively, $\nabla ^2$ is the Laplace–Beltrami operator on $\varOmega$, and *f* and *g* are nonlinear kinetic functions (reaction terms) to be specified. We will consider manifolds with and without boundaries. When boundaries are present, we augment Eq. (1) with the conditions

2 $$\begin{aligned} \varvec{n}\cdot \nabla u = 0, \quad \varvec{n}\cdot \nabla v = 0, \quad \varvec{x} \in \partial \varOmega , \end{aligned}$$

where $\varvec{n}$ is the outward unit normal.

In this paper, we consider patterns defined as stable, time-independent, spatially heterogeneous solutions to Eq. (1). In general, nonlinear reaction–diffusion systems can give rise to a variety of spatiotemporal behaviors, but we restrict our analysis to stationary patterns, which were first predicted to occur by Turing (1952) in the presence of differential diffusion (e.g., $\delta _1 \ne \delta _2)$. In the case of stationary manifolds, conditions can be derived for the instability of spatially homogeneous solutions to Eq. (1) to small perturbations, and these will often lead to patterning. For instance, necessary conditions are given in Murray (2003) as

3a $$\begin{aligned}&f_u + g_v <0, \end{aligned}$$

3b $$\begin{aligned}&f_ug_v - f_vg_u > 0, \end{aligned}$$

3c $$\begin{aligned}&\delta _2 f_u + \delta _1 g_v > 0 ,\end{aligned}$$

3d $$\begin{aligned}&(\delta _2 f_u + \delta _1 g_v )^2 - 4 \delta _1 \delta _2 (f_u g_v - g_u f_v) > 0, \end{aligned}$$

where $f_u,f_v,g_u$, and $g_v$ are the partial derivatives of *f* and *g* with respect to *u* and *v*. Such conditions have been extended to time-dependent domains (Madzvamuse et al. 2010; Klika and Gaffney 2017), as well as to isotropically growing manifolds (Plaza et al. 2004). In these cases, the conditions are more complicated, but the basic idea is the same: differences in the diffusion rates of each species allow for the growth of spatial perturbations to a homogeneous steady state and hence allow for patterning. Here we are primarily concerned with the interactions between growth and the kinds of patterns formed and so will always study cases where diffusion-driven instability occurs in order to elucidate important differences in the kinds of growth. We note that while growth may influence the stability criteria, we intend to always let patterns emerge on a static manifold before initiating growth. In this way, we will use the above conditions to choose parameters within the Turing space such that patterns form before considering the influence of growth on such patterns.

### Reaction–Diffusion Equations on Growing Two-Dimensional Manifolds

We now consider a growing domain in the form of a smooth two-dimensional manifold $\varOmega (t)$ which is simply connected and compact. The manifold $\varOmega (t)$ will either have a smooth boundary $\partial \varOmega (t)$ for all time $t \ge 0$ or will be a manifold without boundary for all time $t \ge 0$. Let $\hat{\varOmega }(t)$ be an area element of the manifold, such that $\hat{\varOmega }(t) \subset \varOmega (t)$. Let $(u,v) : \varOmega (t) \rightarrow \mathbb {R}$ be a concentration function defined on the manifold $\varOmega (t)$, where, for example, $u,\ v$ may describe the concentration of a chemical species, or morphogen, on the manifold $\varOmega (t)$. We shall assume that *u* and *v* are $C^1([0,\infty ))$ in time and $C^2(\varOmega (t))$ in spatial coordinates.

The conservation of mass equation is

4 $$\begin{aligned} \frac{\mathrm{d}}{\mathrm{d}t} \int _{\hat{\varOmega }(t)} u \, \mathrm{d}\varOmega = \int _{\hat{\varOmega }(t)} \left( - \nabla \cdot \mathbf {j} + f(u,v) \right) \mathrm{d}\varOmega , \end{aligned}$$

where $\mathbf {j}$ denotes the flux of concentration *u* and $\mathrm{d}\varOmega$ is the local area element on the manifold. Using Reynold’s transport theorem on the left-hand side of Eq. (4), we have

5 $$\begin{aligned} \frac{\mathrm{d}}{\mathrm{d}t}\int _{\hat{\varOmega }(t)} u \, \mathrm{d}\varOmega = \int _{\hat{\varOmega }(t)} \left( \frac{\partial u}{\partial t} + \nabla _{\varOmega (t)}\cdot (\mathbf {Q}u) \right) \mathrm{d}\varOmega , \end{aligned}$$

where $\mathbf {Q}$ is the local velocity vector field generated by changes in the manifold $\varOmega (t)$ and $\mathrm{d}\varOmega$ is the local area element on the manifold. An analogous equation holds for *v*. Note that we denote $\nabla _{\varOmega (t)}\cdot$ as the divergence operator on $\varOmega (t)$. Similarly, we define $\nabla _{\varOmega (t)}^2$ to be the Laplace–Beltrami operator on $\varOmega (t)$.

By applying Eqs. (4)–(5) and using Fick’s law of diffusion (Plaza et al. 2004), we have the reaction–diffusion–advection system for concentrations *u* and *v* given by:

6a $$\begin{aligned}&\frac{\partial u}{\partial t} + \nabla _{\varOmega (t)} \cdot (\mathbf {Q}u) = \delta _1 \nabla _{\varOmega (t)}^2 u + f(u,v) , \end{aligned}$$

6b $$\begin{aligned}&\frac{\partial v}{\partial t} + \nabla _{\varOmega (t)} \cdot (\mathbf {Q}v) = \delta _2 \nabla _{\varOmega (t)}^2 v + g(u,v) . \end{aligned}$$

Noting that the term $\nabla _{\varOmega (t)} \cdot (\mathbf {Q}u)$ can be written as $\mathbf {Q} \cdot \nabla _{\varOmega (t)} u + u \cdot \nabla _{\varOmega (t)}\mathbf {Q}$, the term $\mathbf {Q} \cdot \nabla _{\varOmega (t)} u$ corresponds to advection due to local growth of the manifold, whereas the $u \cdot \nabla _{\varOmega (t)}\mathbf {Q}$ term corresponds to dilution of the concentration *u* due to local area changes.

### Coordinate Transformation to Stationary Manifolds

In order to solve Eqs. (6a) and (6b), it is often necessary to map the equations to a stationary manifold and solve for *u* and *v* numerically in this stationary domain, before transforming the solved problem back to the original time-dependent manifold. Here, we outline a method to map the evolution equations for *u* and *v* to the stationary manifold $\varPhi \subset \mathbb {R}^2$. We follow a similar procedure to Section 2 of Plaza et al. (2004), however, in a more general form, since we do not insist upon the parametrization for $\varOmega (t)$ to be orthogonal and therefore permit anisotropic growth.

First, parameterize the two-dimensional manifold $\varOmega (t)$ as a surface in $\mathbb {R}^3$ by

7 $$\begin{aligned} \mathbf {X}(\alpha , \beta , t) = \left( \begin{matrix} x(\alpha , \beta , t)\\ y(\alpha , \beta , t)\\ z(\alpha , \beta , t) \end{matrix}\right) , \end{aligned}$$

where $\alpha \in [\alpha _1 , \alpha _2] \subset \mathbb {R}$, $\beta \in [\beta _1, \beta _2] \subset \mathbb {R}$, and $t\ge 0$. As $\varOmega (t)$ is assumed compact, we have that $|\alpha _1|, |\alpha _2|, |\beta _1|, |\beta _2| <\infty$; hence, $(\alpha ,\beta )$ is always confined to a rectangle in $\mathbb {R}^2$. We shall define this set to be the stationary manifold $\varPhi$; in other words, $\varPhi = [\alpha _1, \alpha _2]\times [\beta _1, \beta _2]$. One can always normalize variables so that $|\varPhi | =1$, but this is not necessary.

We shall assume that

8 $$\begin{aligned} \frac{\partial \mathbf {X}}{\partial \alpha } \times \frac{\partial \mathbf {X}}{\partial \beta } \ne \mathbf {0}, \end{aligned}$$

except on a set of measure zero, meaning that $\varOmega (t)$ is a normal surface.

To properly define the Laplace–Beltrami operator, we first define the metric tensor, $\varvec{G}$, by

9 $$\begin{aligned} \varvec{G} = \left( \begin{matrix} g_{11} &{} g_{12}\\ g_{21} &{} g_{22} \end{matrix}\right) , \end{aligned}$$

where

10 $$\begin{aligned} g_{11} = \left| \frac{\partial \mathbf {X}}{\partial \alpha } \right| ^2 , \quad g_{12} = g_{21} = \frac{\partial \mathbf {X}}{\partial \alpha } \cdot \frac{\partial \mathbf {X}}{\partial \beta } , \quad g_{22} = \left| \frac{\partial \mathbf {X}}{\partial \beta } \right| ^2 . \end{aligned}$$

The determinant of the metric tensor is

11 $$\begin{aligned} \det \varvec{G} = g_{11}g_{22} - g_{12}^2 = \left| \frac{\partial \mathbf {X}}{\partial \alpha } \right| ^2 \left| \frac{\partial \mathbf {X}}{\partial \beta } \right| ^2 - \left( \frac{\partial \mathbf {X}}{\partial \alpha } \cdot \frac{\partial \mathbf {X}}{\partial \beta } \right) ^2, \end{aligned}$$

while the inverse of the metric tensor, $\varvec{G}^{-1}$, is defined by

12 $$\begin{aligned} \varvec{G}^{-1} = \frac{1}{\det \varvec{G}}\left( \begin{matrix} g_{22} &{} -g_{12}\\ -g_{12} &{} g_{11} \end{matrix}\right) = \frac{1}{g_{11}g_{22} - g_{12}^2}\left( \begin{matrix} g_{22} &{} -g_{12}\\ -g_{12} &{} g_{11} \end{matrix}\right) . \end{aligned}$$

The representation of the Laplace–Beltrami operator $\nabla _{\varOmega (t)}^2$ on the stationary two-dimensional manifold $\varPhi$ is then given by

13 $$\begin{aligned} \nabla _{\varOmega (t)}^2= & {} \frac{1}{|\det \varvec{G}|^{1/2}}\left\{ \frac{\partial }{\partial \alpha }\left( |\det \varvec{G}|^{1/2} \varvec{G}_{11}^{-1}\frac{\partial }{\partial \alpha } \right) + \frac{\partial }{\partial \alpha }\left( |\det \varvec{G}|^{1/2} \varvec{G}_{12}^{-1}\frac{\partial }{\partial \beta } \right) \right. \nonumber \\&+ \left. \frac{\partial }{\partial \beta }\left( |\det \varvec{G}|^{1/2} \varvec{G}_{21}^{-1}\frac{\partial }{\partial \alpha } \right) + \frac{\partial }{\partial \beta }\left( |\det \varvec{G}|^{1/2} \varvec{G}_{22}^{-1}\frac{\partial }{\partial \beta } \right) \right\} .\nonumber \\ \end{aligned}$$

In the case of a diagonal metric tensor (as seen in isotropic growth), note that $\varvec{G}_{12}^{-1} = \varvec{G}_{21}^{-1} =0$, and hence, expression Eq. (13) simplifies to a form like that given in Eq. (14) of Plaza et al. (2004).

In order to remove the advection term induced by the growing manifold [the $\nabla _{\varOmega (t)} \cdot (\mathbf {Q}u)$ terms in Eq. (6)], we shall apply a change of variable akin to that used in Crampin et al. (1999) and Plaza et al. (2004). We shall assume that space and time variables in $\mathbf {X}$ are separable. Noting that

14 $$\begin{aligned} \nabla _{\varOmega (t)} \cdot (\mathbf {Q}u) = (\nabla _{\varOmega (t)}\cdot \mathbf {Q})u + \mathbf {Q}\cdot (\nabla _{\varOmega (t)}u), \end{aligned}$$

the change of variable will contribute a term $-\mathbf {Q}\cdot (\nabla _{\varOmega (t)}u)$, canceling the latter term in Eq. (14). Then, after the coordinate change, we will have a contributing advection term of the form $(\nabla _{\varOmega (t)}\cdot \mathbf {Q})u$. The form of the divergence of the advection vector generated due to the change in $\varOmega (t)$ is intrinsic to $\varOmega (t)$ and may be calculated by the coordinate change $\mathbf {X}$ using again the metric tensor. We find

15 $$\begin{aligned} \nabla _{\varOmega (t)}\cdot \mathbf {Q} = \frac{\partial }{\partial t}\left( \log \left( |\det \varvec{G}|^{1/2}\right) \right) . \end{aligned}$$

In the case where the metric tensor is diagonal (for instance, as occurs for isotropic growth), the formula above reduces to

16 $$\begin{aligned} \nabla _{\varOmega (t)}\cdot \mathbf {Q} = \frac{\partial }{\partial t}\left( \log \left( \left| \frac{\partial \mathbf {X}}{\partial \alpha } \right| \left| \frac{\partial \mathbf {X}}{\partial \beta } \right| \right) \right) , \end{aligned}$$

which is equivalent to that given in Eq. (13) of Plaza et al. (2004).

### Reaction–Diffusion System on the Stationary Manifold

Using the form of the Laplace–Beltrami operator given by Eq. (13) and the advection term given by Eq. (15), then we can write reaction–diffusion system Eq. (6) on the growing manifold $\varOmega (t)$ as a spatially and temporally heterogeneous reaction–diffusion system on the stationary manifold $\varPhi$, obtaining

17a $$\begin{aligned} \frac{\partial u}{\partial t}= & {} \frac{1}{|\det \varvec{G}|^{1/2}}\left\{ \frac{\partial }{\partial \alpha }\left( |\det \varvec{G}|^{1/2}\varvec{G}_{11}^{-1}\frac{\partial u}{\partial \alpha } \right) + \frac{\partial }{\partial \alpha }\left( |\det \varvec{G}|^{1/2}\varvec{G}_{12}^{-1}\frac{\partial u}{\partial \beta } \right) \right. \nonumber \\&+\, \left. \frac{\partial }{\partial \beta }\left( |\det \varvec{G}|^{1/2}\varvec{G}_{21}^{-1}\frac{\partial u}{\partial \alpha } \right) + \frac{\partial }{\partial \beta }\left( |\det \varvec{G}|^{1/2}\varvec{G}_{22}^{-1}\frac{\partial u}{\partial \beta } \right) \right\} \nonumber \\&-\, \frac{\partial }{\partial t}\left( \log \left( |\det \varvec{G}|^{1/2}\right) \right) u + f(u,v), \end{aligned}$$

17b $$\begin{aligned} \frac{\partial v}{\partial t}= & {} \frac{1}{|\det \varvec{G}|^{1/2}}\left\{ \frac{\partial }{\partial \alpha }\left( |\det \varvec{G}|^{1/2}\varvec{G}_{11}^{-1}\frac{\partial v}{\partial \alpha } \right) + \frac{\partial }{\partial \alpha }\left( |\det \varvec{G}|^{1/2}\varvec{G}_{12}^{-1}\frac{\partial v}{\partial \beta } \right) \right. \nonumber \\&+\, \left. \frac{\partial }{\partial \beta }\left( |\det \varvec{G}|^{1/2}\varvec{G}_{21}^{-1}\frac{\partial v}{\partial \alpha } \right) + \frac{\partial }{\partial \beta }\left( |\det \varvec{G}|^{1/2}\varvec{G}_{22}^{-1}\frac{\partial v}{\partial \beta } \right) \right\} \nonumber \\&-\, \frac{\partial }{\partial t}\left( \log \left( |\det \varvec{G}|^{1/2}\right) \right) v + g(u,v). \end{aligned}$$

### Examples of Flat and Curved Domains

We now define a pair of two-dimensional manifolds which we will use as examples for isotropic and anisotropic growth: a planar ellipse and the surface of an ellipsoid. There are many biologically relevant manifolds one could study, but these examples are sufficient to compare the influence of isotropic and anisotropic growth, and compare effects due to (boundary) curvature of the manifold.

#### Planar Ellipse

We parameterize a growing ellipse in the plane via the semimajor and semiminor axes, given by *A*(*t*) and *B*(*t*), respectively. These must satisfy

18 $$\begin{aligned} \dfrac{x^2}{A^2(t)} + \dfrac{y^2}{B^2(t)} \le 1. \end{aligned}$$

We define the surface parametrically via

19 $$\begin{aligned} \mathbf {X}(\alpha ,\beta ,t) = \left( \begin{matrix} A(t) \alpha \cos (\beta )\\ B(t) \alpha \sin (\beta )\\ 0 \end{matrix}\right) , \end{aligned}$$

where $\alpha \in [0,1]$ and $\beta \in [0,2\pi )$. We calculate

20 $$\begin{aligned}&\frac{\partial \mathbf {X}}{\partial \alpha } = \left( \begin{matrix} A(t) \cos (\beta )\\ B(t) \sin (\beta )\\ 0 \end{matrix}\right) , \quad \frac{\partial \mathbf {X}}{\partial \beta } = \left( \begin{matrix} -A(t) \alpha \sin (\beta )\\ B(t) \alpha \cos (\beta )\\ 0 \end{matrix}\right) , \quad \nonumber \\&\frac{\partial \mathbf {X}}{\partial \alpha } \times \frac{\partial \mathbf {X}}{\partial \beta } = \left( \begin{matrix} 0\\ 0\\ A(t)B(t)\alpha \end{matrix}\right) . \end{aligned}$$

The entries of the metric tensor $\varvec{G}$ are given by

21a $$\begin{aligned} g_{11}= & {} \left| \frac{\partial \mathbf {X}}{\partial \alpha } \right| ^2 = A^2 \cos ^2(\beta ) + B^2 \sin ^2(\beta ) , \end{aligned}$$

21b $$\begin{aligned} g_{12}= & {} g_{21} = \frac{\partial \mathbf {X}}{\partial \alpha } \cdot \frac{\partial \mathbf {X}}{\partial \beta } = (B^2-A^2)\alpha \sin (\beta )\cos (\beta ), \end{aligned}$$

21c $$\begin{aligned} g_{22}= & {} \left| \frac{\partial \mathbf {X}}{\partial \beta } \right| ^2 = \left( A^2 \sin ^2(\beta ) + B^2 \cos ^2(\beta ) \right) \alpha ^2 . \end{aligned}$$

The determinant of the metric tensor then reads

22 $$\begin{aligned} \det \varvec{G} = g_{11}g_{22} - g_{12}^2 = A^2B^2 \alpha ^2. \end{aligned}$$

Substituting the above into the formulae for Laplace–Beltrami operator Eq. (13) and for advective growth term Eq. (15), we find after simplifying

23 $$\begin{aligned} \nabla _{\varOmega (t)}^2&= \frac{1}{AB\alpha }\frac{\partial }{\partial \alpha }\left( \frac{g_{22}}{AB\alpha }\frac{\partial }{\partial \alpha } \right) - \frac{1}{AB\alpha }\frac{\partial }{\partial \alpha }\left( \frac{g_{12}}{AB\alpha }\frac{\partial }{\partial \beta } \right) \nonumber \\&\quad - \frac{1}{AB\alpha }\frac{\partial }{\partial \beta }\left( \frac{g_{12}}{AB\alpha }\frac{\partial }{\partial \alpha } \right) + \frac{1}{AB\alpha }\frac{\partial }{\partial \beta }\left( \frac{g_{11}}{AB\alpha }\frac{\partial }{\partial \beta } \right) , \nonumber \\&= \frac{1}{A^2B^2\alpha }\left\{ \frac{\partial }{\partial \alpha }\left( \left( A^2\sin ^2\beta + B^2 \cos ^2(\beta )\right) \alpha \frac{\partial }{\partial \alpha } \right) + \frac{\partial }{\partial \alpha }\left( \left( A^2 - B^2\right) \sin (\beta )\cos (\beta ) \frac{\partial }{\partial \beta } \right) \right. \nonumber \\&\quad + \left. \frac{\partial }{\partial \beta }\left( \left( A^2 - B^2\right) \sin (\beta )\cos (\beta ) \frac{\partial }{\partial \alpha } \right) + \frac{\partial }{\partial \beta }\left( \frac{A^2\cos ^2(\beta ) + B^2\sin ^2(\beta )}{\alpha } \frac{\partial }{\partial \theta } \right) \right\} , \nonumber \\&= \frac{A^2\sin ^2(\beta )+B^2\cos ^2(\beta )}{A^2B^2}\left( \frac{\partial ^2}{\partial \alpha ^2} + \frac{1}{\alpha }\frac{\partial }{\partial \alpha } \right) + \frac{A^2\cos ^2(\beta ) + B^2\sin ^2(\beta )}{A^2B^2\alpha ^2}\frac{\partial ^2}{\partial \beta ^2} \nonumber \\&\quad + \frac{2\left( A^2-B^2\right) }{A^2B^2\alpha }\frac{\partial ^2}{\partial \alpha \partial \beta } + \frac{A^2 -B^2}{A^2B^2}\left( \frac{\cos ^2(\beta )-\sin ^2(\beta )}{\alpha }\right) \frac{\partial }{\partial \alpha } \nonumber \\&\quad + \frac{\left( B^2-A^2\right) \sin (\beta )\cos (\beta )}{A^2B^2\alpha }\frac{\partial }{\partial \beta }, \end{aligned}$$

and

24 $$\begin{aligned} \nabla _{\varOmega (t)}\cdot \mathbf {Q} = \frac{\partial }{\partial t}\left( \log \left( |\det G|^{1/2}\right) \right) = \frac{\partial }{\partial t}\left( \log (A) + \log (B) + \log (\alpha )\right) = \frac{\dot{A}}{A}+\frac{\dot{B}}{B},\nonumber \\ \end{aligned}$$

where we denote derivatives of $A(t), \, B(t)$ with respect to time as $\dot{A}=\frac{\mathrm{d}A}{\mathrm{d}t}$ and $\dot{B}=\frac{\mathrm{d}B}{\mathrm{d}t}$. System Eq. (6) is then put into the form

25a $$\begin{aligned} \frac{\partial u}{\partial t}= & {} \delta _1\frac{A^2\sin ^2(\beta )+B^2\cos ^2(\beta )}{A^2B^2}\left( \frac{\partial ^2 u}{\partial \alpha ^2} + \frac{1}{\alpha }\frac{\partial u}{\partial \alpha } \right) + \delta _1\frac{A^2\cos ^2(\beta ) + B^2\sin ^2(\beta )}{A^2B^2\alpha ^2}\frac{\partial ^2 u}{\partial \beta ^2}\nonumber \\&+\, \delta _1\frac{2\left( A^2-B^2\right) }{A^2B^2\alpha }\frac{\partial ^2 u}{\partial \alpha \partial \beta } + \delta _1\frac{A^2 -B^2}{A^2B^2}\left( \frac{\cos ^2(\beta )-\sin ^2(\beta )}{\alpha }\right) \frac{\partial u}{\partial \alpha } \nonumber \\&+\,\delta _1\frac{\left( B^2-A^2\right) \sin (\beta )\cos (\beta )}{A^2B^2\alpha }\frac{\partial u}{\partial \beta } - \left( \frac{\dot{A}}{A}+\frac{\dot{B}}{B}\right) u + f(u,v), \end{aligned}$$

25b $$\begin{aligned} \frac{\partial v}{\partial t}= & {} \delta _2\frac{A^2\sin ^2(\beta )+B^2\cos ^2(\beta )}{A^2B^2}\left( \frac{\partial ^2 v}{\partial \alpha ^2} + \frac{1}{\alpha }\frac{\partial v}{\partial \alpha } \right) + \delta _2\frac{A^2\cos ^2(\beta ) + B^2\sin ^2(\beta )}{A^2B^2\alpha ^2}\frac{\partial ^2 v}{\partial \beta ^2}\nonumber \\&+\, \delta _2\frac{2\left( A^2-B^2\right) }{A^2B^2\alpha }\frac{\partial ^2 v}{\partial \alpha \partial \beta } + \delta _2\frac{A^2 -B^2}{A^2B^2}\left( \frac{\cos ^2(\beta )-\sin ^2(\beta )}{\alpha }\right) \frac{\partial v}{\partial \alpha } \nonumber \\&+\, \delta _2\frac{\left( B^2-A^2\right) \sin (\beta )\cos (\beta )}{A^2B^2\alpha }\frac{\partial v}{\partial \beta } - \left( \frac{\dot{A}}{A}+\frac{\dot{B}}{B}\right) v + g(u,v). \end{aligned}$$

We note that in the case where $A(t)\equiv B(t)$ (an isotropically growing circular disk), the system above reduces to

26a $$\begin{aligned} \frac{\partial u}{\partial t}= & {} \frac{\delta _1}{A^2}\left( \frac{\partial ^2 u}{\partial \alpha ^2} + \frac{1}{\alpha }\frac{\partial u}{\partial \alpha } + \frac{1}{\alpha ^2}\frac{\partial ^2 u}{\partial \beta ^2} \right) - 2\frac{\dot{A}}{A} u + f(u,v), \end{aligned}$$

26b $$\begin{aligned} \frac{\partial v}{\partial t}= & {} \frac{\delta _2}{A^2}\left( \frac{\partial ^2 v}{\partial \alpha ^2} + \frac{1}{\alpha }\frac{\partial u}{\partial \alpha } + \frac{1}{\alpha ^2}\frac{\partial ^2 v}{\partial \beta ^2}\right) - 2\frac{\dot{A}}{A} v + g(u,v). \end{aligned}$$

This is the same as given for isotropic growth of a disk in Eq. (34) of Plaza et al. (2004).

#### Growing Tri-Axial Ellipsoid Surface

Another manifold we consider is the surface of a growing tri-axial ellipsoid. Murray (2003) discusses reaction–diffusion models as a mechanism for spiral patterning on amphibian egg shells. Since the shape of these eggs may be approximated by an ellipsoid, studying reaction–diffusion systems on these sorts of domains could help predict the patterns formed due to the calcium waves diffusing over the surface of the shell.

In order to consider reaction–diffusion system Eq. (6) on the surface of the growing tri-axial ellipsoid, we follow the same analysis as outlined in Sect. 2.3. For the growing tri-axial ellipsoid,

27 $$\begin{aligned} \dfrac{x^2}{A^2(t)} + \dfrac{y^2}{B^2(t)} + \dfrac{z^2}{C^2(t)} = 1, \end{aligned}$$

we define the surface parametrically via

28 $$\begin{aligned} \mathbf {X}(\alpha ,\beta ,t) = \left( \begin{matrix} A(t) \sin (\alpha ) \cos (\beta )\\ B(t) \sin (\alpha ) \sin (\beta )\\ C(t) \cos (\alpha ) \end{matrix}\right) , \end{aligned}$$

where $\alpha \in [0,\pi ]$ and $\beta \in [0,2\pi )$ are the polar and azimuthal angles, respectively. Then, we calculate

29 $$\begin{aligned}&\frac{\partial \mathbf {X}}{\partial \alpha } = \left( \begin{matrix} A(t) \cos (\alpha )\cos (\beta )\\ B(t) \cos (\alpha )\sin (\beta )\\ -C(t)\sin (\alpha ) \end{matrix}\right) , \end{aligned}$$

30 $$\begin{aligned}&\frac{\partial \mathbf {X}}{\partial \beta } = \left( \begin{matrix} -A(t) \sin (\alpha ) \sin (\beta )\\ B(t)\sin (\alpha ) \cos (\beta )\\ 0 \end{matrix}\right) , \end{aligned}$$

31 $$\begin{aligned}&\frac{\partial \mathbf {X}}{\partial \alpha } \times \frac{\partial \mathbf {X}}{\partial \beta } = \left( \begin{matrix} B(t)C(t)\sin ^2(\alpha )\cos (\beta )\\ A(t)C(t) \sin ^2(\alpha )\sin (\beta )\\ A(t)B(t)\cos (\alpha )\sin (\alpha ) \end{matrix}\right) . \end{aligned}$$

Calculating the entries of the metric tensor $\varvec{G}$, we find these to be

32 $$\begin{aligned}&g_{11} = \left| \frac{\partial \mathbf {X}}{\partial \alpha } \right| ^2 = \left( A^2 \cos ^2(\beta ) + B^2 \sin ^2(\beta )\right) \cos ^2(\alpha ) + C^2\sin ^2(\alpha ) , \end{aligned}$$

33 $$\begin{aligned}&g_{12} = g_{21} = \frac{\partial \mathbf {X}}{\partial \alpha } \cdot \frac{\partial \mathbf {X}}{\partial \beta } = \big (B^2-A^2 \big ) \sin (\alpha )\cos (\alpha ) \sin (\beta )\cos (\beta ), \end{aligned}$$

34 $$\begin{aligned}&g_{22} = \left| \frac{\partial \mathbf {X}}{\partial \beta } \right| ^2 = \left( A^2 \sin ^2(\beta ) + B^2 \cos ^2(\beta ) \right) \sin ^2(\alpha ) . \end{aligned}$$

Thus, we find the determinant of the metric tensor $\varvec{G}$ to be

35 $$\begin{aligned} \det \varvec{G}= & {} g_{11}g_{22} - g_{12}^2 \nonumber \\= & {} \left\{ A^2B^2 \cos ^2(\alpha ) + C^2 \left( A^2\sin ^2(\beta ) + B^2\cos ^2(\beta ) \right) \sin ^2(\alpha ) \right\} \sin ^2(\alpha ).\quad \quad \end{aligned}$$

Substituting these expressions into Laplace–Beltrami operator Eq. (13) we find that

36 $$\begin{aligned} \nabla _{\varOmega (t)}^2= & {} \dfrac{A^2\sin ^2(\beta ) + B^2\cos ^2(\beta ) }{ A^2B^2 \cos ^2(\alpha ) + C^2 \left( A^2\sin ^2(\beta ) + B^2\cos ^2(\beta ) \right) \sin ^2(\alpha )}\dfrac{\partial ^2}{\partial \alpha ^2}\nonumber \\&+ \dfrac{\left( A^2 \cos ^2(\beta ) + B^2 \sin ^2(\beta )\right) \cos ^2(\alpha ) + C^2\sin ^2(\alpha ) }{\left\{ A^2B^2 \cos ^2(\alpha ) + C^2 \left( A^2\sin ^2(\beta ) + B^2\cos ^2(\beta ) \right) \sin ^2(\alpha ) \right\} \sin ^2(\alpha )}\dfrac{\partial ^2}{\partial \beta ^2}\nonumber \\&- \dfrac{2(B^2-A^2) \sin (\alpha )\cos (\alpha ) \sin (\beta )\cos (\beta )}{\left\{ A^2B^2 \cos ^2(\alpha ) + C^2 \left( A^2\sin ^2(\beta ) + B^2\cos ^2(\beta ) \right) \sin ^2(\alpha ) \right\} \sin ^2(\alpha )}\dfrac{\partial ^2}{\partial \alpha \partial \beta }\nonumber \\&+ \dfrac{M_1(\alpha ,\beta )}{\left( \left\{ A^2B^2 \cos ^2(\alpha ) + C^2 \left( A^2\sin ^2(\beta ) + B^2\cos ^2(\beta ) \right) \sin ^2(\alpha ) \right\} \sin ^2(\alpha )\right) ^2}\dfrac{\partial }{\partial \alpha }\nonumber \\&+ \dfrac{M_2(\alpha ,\beta )}{\left( \left\{ A^2B^2 \cos ^2(\alpha ) + C^2 \left( A^2\sin ^2(\beta ) + B^2\cos ^2(\beta ) \right) \sin ^2(\alpha ) \right\} \sin ^2(\alpha )\right) ^2}\dfrac{\partial }{\partial \beta },\nonumber \\ \end{aligned}$$

where the functions $M_1$ and $M_2$ are defined by

37 $$\begin{aligned} M_1(\alpha ,\beta )= & {} \cos (\alpha )\sin ^3(\alpha )\left\{ A^2B^2\left[ \left( A^2\sin ^2(\beta ) +B^2\cos ^2(\beta )\right) \sin ^2(\alpha ) \right. \right. \nonumber \\&\left. + \left( A^2\cos ^2(\beta ) +B^2\sin ^2(\beta )\right) \cos ^2(\alpha ) \right] \nonumber \\&\left. + C^2\left( A^2 - B^2 \right) \left( B^2\cos ^4(\beta )-A^2\sin ^4(\beta )\right) \right\} , \end{aligned}$$

and

38 $$\begin{aligned} M_2(\alpha ,\beta )= & {} \left( B^2 - A^2\right) \sin (\beta )\cos (\beta )\left\{ 2A^2B^2\cos ^4(\alpha ) \right. \nonumber \\&\left. + C^2\left( 2\sin ^4(\alpha )+3\cos ^2(\alpha )\right) \left( A^2\sin ^2(\beta ) + B^2\cos ^2(\beta ) \right) \right\} . \end{aligned}$$

Further, substituting the information from the metric tensor $\varvec{G}$ into advective growth term Eq. (15), we have

39 $$\begin{aligned} \nabla _{\varOmega (t)}\cdot \mathbf {Q}= & {} \frac{\partial }{\partial t}\left( \log \left( |\det \varvec{G}|^{1/2}\right) \right) \,\nonumber \\= & {} \frac{1}{2}\frac{\partial }{\partial t} \log \left( A^2B^2 \cos ^2(\alpha ) + C^2 \left( A^2\sin ^2(\beta ) + B^2\cos ^2(\beta ) \right) \sin ^2(\alpha ) \right) \,\nonumber \\= & {} \dfrac{ A^2B^2\gamma _{AB}(t) \cos ^2(\alpha ) + C^2 \left( A^2 \gamma _{AC}(t) \sin ^2(\beta ) + B^2 \gamma _{BC}(t)\cos ^2(\beta ) \right) \sin ^2(\alpha )}{A^2B^2 \cos ^2(\alpha ) + C^2 \left( A^2\sin ^2(\beta ) + B^2\cos ^2(\beta ) \right) \sin ^2(\alpha )},\nonumber \\ \end{aligned}$$

where we have defined

40a $$\begin{aligned}&\gamma _{AB}(t) = \frac{\mathrm{d}}{\mathrm{d}t}\log (AB) = \frac{\dot{A}}{A} + \frac{\dot{B}}{B}, \end{aligned}$$

40b $$\begin{aligned}&\gamma _{AC}(t) = \frac{\mathrm{d}}{\mathrm{d}t}\log (AC) = \frac{\dot{A}}{A} + \frac{\dot{C}}{C}, \end{aligned}$$

40c $$\begin{aligned}&\gamma _{BC}(t) = \frac{\mathrm{d}}{\mathrm{d}t}\log (BC) = \frac{\dot{B}}{B} + \frac{\dot{C}}{C}, \end{aligned}$$

for ease of notation. We denote the derivatives of $A(t), \, B(t),$ and *C*(*t*) with respect to time as $\dot{A} = \frac{\mathrm{d}A}{\mathrm{d}t},\, \dot{B} = \frac{\mathrm{d}B}{\mathrm{d}t},$ and $\dot{C} = \frac{\mathrm{d}C}{\mathrm{d}t}$, respectively.

Then, reaction–diffusion system Eq. (6) can be put into the form

41a $$\begin{aligned} \dfrac{\partial u}{\partial t}= & {} \delta _1\frac{A^2\sin ^2(\beta ) + B^2\cos ^2(\beta ) }{ A^2B^2 \cos ^2(\alpha ) + C^2 \left( A^2\sin ^2(\beta ) + B^2\cos ^2(\beta ) \right) \sin ^2(\alpha )}\dfrac{\partial ^2 u}{\partial \alpha ^2}\nonumber \\&+\, \delta _1\dfrac{\left( A^2 \cos ^2(\beta ) + B^2 \sin ^2(\beta )\right) \cos ^2(\alpha ) + C^2\sin ^2(\alpha ) }{\left\{ A^2B^2 \cos ^2(\alpha ) + C^2 \left( A^2\sin ^2(\beta ) + B^2\cos ^2(\beta ) \right) \sin ^2(\alpha ) \right\} \sin ^2(\alpha )}\dfrac{\partial ^2 u}{\partial \beta ^2}\nonumber \\&-\, \delta _1 \dfrac{2(B^2-A^2) \sin (\alpha )\cos (\alpha ) \sin (\beta )\cos (\beta )}{\left\{ A^2B^2 \cos ^2(\alpha ) + C^2 \left( A^2\sin ^2(\beta ) + B^2\cos ^2(\beta ) \right) \sin ^2(\alpha ) \right\} \sin ^2(\alpha )}\dfrac{\partial ^2 u}{\partial \alpha \partial \beta }\nonumber \\&+\, \delta _1 \dfrac{M_1(\alpha ,\beta )}{\left( \left\{ A^2B^2 \cos ^2(\alpha ) + C^2 \left( A^2\sin ^2(\beta ) + B^2\cos ^2(\beta ) \right) \sin ^2(\alpha ) \right\} \sin ^2(\alpha )\right) ^2}\dfrac{\partial u}{\partial \alpha }\nonumber \\&+\, \delta _1 \dfrac{M_2(\alpha ,\beta )}{\left( \left\{ A^2B^2 \cos ^2(\alpha ) + C^2 \left( A^2\sin ^2(\beta ) + B^2\cos ^2(\beta ) \right) \sin ^2(\alpha ) \right\} \sin ^2(\alpha )\right) ^2}\dfrac{\partial u}{\partial \beta } \nonumber \\&-\,\dfrac{ A^2B^2\gamma _{AB}(t) \cos ^2(\alpha ) + C^2 \left( A^2 \gamma _{AC}(t) \sin ^2(\beta ) + B^2 \gamma _{BC}(t)\cos ^2(\beta ) \right) \sin ^2(\alpha )}{A^2B^2 \cos ^2(\alpha ) + C^2 \left( A^2\sin ^2(\beta ) + B^2\cos ^2(\beta ) \right) \sin ^2(\alpha )} u \nonumber \\&+\, f(u,v), \end{aligned}$$

41b $$\begin{aligned} \frac{\partial v}{\partial t}= & {} \delta _2\dfrac{A^2\sin ^2(\beta ) + B^2\cos ^2(\beta ) }{ A^2B^2 \cos ^2(\alpha ) + C^2 \left( A^2\sin ^2(\beta ) + B^2\cos ^2(\beta ) \right) \sin ^2(\alpha )}\frac{\partial ^2 v}{\partial \alpha ^2}\nonumber \\&+\, \delta _2\dfrac{\left( A^2 \cos ^2(\beta ) + B^2 \sin ^2(\beta )\right) \cos ^2(\alpha ) + C^2\sin ^2(\alpha ) }{\left\{ A^2B^2 \cos ^2(\alpha ) + C^2 \left( A^2\sin ^2(\beta ) + B^2\cos ^2(\beta ) \right) \sin ^2(\alpha ) \right\} \sin ^2(\alpha )}\dfrac{\partial ^2 v}{\partial \beta ^2}\nonumber \\&-\, \delta _2 \dfrac{2(B^2-A^2) \sin (\alpha )\cos (\alpha ) \sin (\beta )\cos (\beta )}{\left\{ A^2B^2 \cos ^2(\alpha ) + C^2 \left( A^2\sin ^2(\beta ) + B^2\cos ^2(\beta ) \right) \sin ^2(\alpha ) \right\} \sin ^2(\alpha )}\dfrac{\partial ^2 v}{\partial \alpha \partial \beta }\nonumber \\&+\, \delta _2 \dfrac{M_1(\alpha ,\beta )}{\left( \left\{ A^2B^2 \cos ^2(\alpha ) + C^2 \left( A^2\sin ^2(\beta ) + B^2\cos ^2(\beta ) \right) \sin ^2(\alpha ) \right\} \sin ^2(\alpha )\right) ^2}\dfrac{\partial v}{\partial \alpha }\nonumber \\&+\, \delta _2 \dfrac{M_2(\alpha ,\beta )}{\left( \left\{ A^2B^2 \cos ^2(\alpha ) + C^2 \left( A^2\sin ^2(\beta ) + B^2\cos ^2(\beta ) \right) \sin ^2(\alpha ) \right\} \sin ^2(\alpha )\right) ^2}\dfrac{\partial v}{\partial \beta } \nonumber \\&-\,\dfrac{ A^2B^2\gamma _{AB}(t) \cos ^2(\alpha ) + C^2 \left( A^2 \gamma _{AC}(t) \sin ^2(\beta ) + B^2 \gamma _{BC}(t)\cos ^2(\beta ) \right) \sin ^2(\alpha )}{A^2B^2 \cos ^2(\alpha ) + C^2 \left( A^2\sin ^2(\beta ) + B^2\cos ^2(\beta ) \right) \sin ^2(\alpha )} v \nonumber \\&+\, g(u,v). \end{aligned}$$

In the case where $A(t)\equiv B(t) \equiv C(t)$ (i.e., where the domain is a growing spherical surface under isotropic growth), the reaction–diffusion system above reduces to

42a $$\begin{aligned} \dfrac{\partial u}{\partial t}= & {} \dfrac{\delta _1}{A^2}\left( \dfrac{\partial ^2 u}{\partial \alpha ^2} + \dfrac{\cos (\alpha )}{\sin (\alpha )}\dfrac{\partial u}{\partial \alpha } + \dfrac{1}{\sin ^2(\alpha )}\dfrac{\partial ^2 u}{\partial \beta ^2} \right) - 2\frac{\dot{A}}{A} u + f(u,v), \end{aligned}$$

42b $$\begin{aligned} \dfrac{\partial v}{\partial t}= & {} \dfrac{\delta _2}{A^2}\left( \dfrac{\partial ^2 v}{\partial \alpha ^2} + \dfrac{\cos (\alpha )}{\sin (\alpha )}\dfrac{\partial v}{\partial \alpha } + \dfrac{1}{\sin ^2(\alpha )}\dfrac{\partial ^2 v}{\partial \beta ^2} \right) - 2\frac{\dot{A}}{A} v + g(u,v). \end{aligned}$$

In the special case of a spheroid, $A(t) \equiv B(t)$ with $A(t) \ne C(t)$, reaction–diffusion system Eq. (41) reduces to

43a $$\begin{aligned} \dfrac{\partial u}{\partial t}= & {} \dfrac{\delta _1}{A^2}\left( \dfrac{1}{A^2\cos ^2(\alpha )+C^2\sin ^2(\alpha )}\dfrac{\partial ^2 u}{\partial \alpha ^2} \right. \nonumber \\&\left. + \dfrac{A^4}{\left( A^2\cos ^2(\alpha )+C^2\sin ^2(\alpha )\right) ^2} \dfrac{\cos (\alpha )}{\sin (\alpha )}\dfrac{\partial u}{\partial \alpha } + \dfrac{1}{\sin ^2(\alpha )}\dfrac{\partial ^2 u}{\partial \beta ^2} \right) \nonumber \\&- \dfrac{2A\dot{A}\cos ^2(\alpha ) + C^2 \left( \frac{\dot{A}}{A} + \frac{\dot{C}}{C}\right) \sin ^2(\alpha )}{A^2\cos ^2(\alpha )+C^2\sin ^2(\alpha )} u + f(u,v), \end{aligned}$$

43b $$\begin{aligned} \dfrac{\partial v}{\partial t}= & {} \dfrac{\delta _2}{A^2}\left( \dfrac{1}{A^2\cos ^2(\alpha )+C^2\sin ^2(\alpha )}\dfrac{\partial ^2 v}{\partial \alpha ^2} \right. \nonumber \\&\left. + \dfrac{A^4}{\left( A^2\cos ^2(\alpha )+C^2\sin ^2(\alpha )\right) ^2} \dfrac{\cos (\alpha )}{\sin (\alpha )}\dfrac{\partial v}{\partial \alpha } + \dfrac{1}{\sin ^2(\alpha )}\dfrac{\partial ^2 v}{\partial \beta ^2} \right) \nonumber \\&- \dfrac{2A\dot{A}\cos ^2(\alpha ) + C^2 \left( \frac{\dot{A}}{A} + \frac{\dot{C}}{C}\right) \sin ^2(\alpha )}{A^2\cos ^2(\alpha )+C^2\sin ^2(\alpha )} v + g(u,v). \end{aligned}$$

## Methods for the Study of Growth Rates, Curvature, and Anisotropic Growth

In this section, we will describe the various kinds of reaction kinetics that we have explored, as well as our systematic approach to studying them on growing domains. We are chiefly interested in fully nonlinear and stationary patterns, rather than transient patterning, so we always allow the reaction–diffusion process to settle onto a steady state on a stationary manifold before and after it has undergone growth. In this way, we can compare the kinds of stationary patterns which are arrived at via different growth processes. We will proceed to discuss parameter choices and numerical methods which will be used in our analysis.

### Types of Reaction Kinetics

There are a multitude of functions $f, \ g$ which can represent the interaction between two morphogens. We consider three different reaction schemes: Schnakenberg, Gierer–Meinhardt, and FitzHugh–Nagumo. These are canonical examples of cross- and pure activator–inhibitor kinetics, as well as excitable media, respectively.

#### Schnakenberg Reaction Kinetics

First, we consider Schnakenberg (also known as activator-depleted) kinetics, a well-studied form of reaction which models activator–inhibitor behavior (Schnakenberg 1979). They were originally developed to explore simple chemical kinetics that gave rise to oscillatory behavior, but they have since been used in a huge amount of literature as a canonical example of activator–inhibitor chemistry (Iron et al. 2004; Liu et al. 2013; Sarfaraz and Madzvamuse 2017).

Taking the non-dimensionalized form of the Schnakenberg kinetic system as in Krause et al. (2018a), the reaction terms $f, \ g$ in Eq. (1) are given by

44a $$\begin{aligned}&f(u,v) = a - u + u^2v, \end{aligned}$$

44b $$\begin{aligned}&g(u,v) = b - u^2v, \end{aligned}$$

where *a* and *b* are parameters to be chosen. In order for patterns to form, the parameters $a, \ b, \ \delta _1, \ \delta _2$ must satisfy Turing instability condition Eq. (3) given in Sect. 2.1. Following the methodology outlined in Sect. 2.1, we first find the homogeneous steady state of the system by setting $f= 0 = g$. This gives the steady-state $\mathbf {u}^*$ and the Jacobian of the linearized system $\varvec{J}^*$ as

45 $$\begin{aligned} \mathbf {u}^* = \begin{pmatrix} a+ b,&\dfrac{b}{(a+b)^2} \end{pmatrix} , \qquad \varvec{J}^* = \begin{pmatrix} \dfrac{b-a}{a+b} &{} (a+b)^2 \\ \\ \dfrac{-2b}{a+b} &{} - (a+b)^2 \end{pmatrix}. \end{aligned}$$

Turing instability condition Eqs. (3) can be written as:

46a $$\begin{aligned}&(a+b)^3 - (b-a) >0 , \end{aligned}$$

46b $$\begin{aligned}&a + b >0, \end{aligned}$$

46c $$\begin{aligned}&\delta _2 (b - a) - \delta _1 (a+b)^3 >0, \end{aligned}$$

46d $$\begin{aligned}&\left( \delta _2 (b - a) - \delta _1 (a+b)^3 \right) ^2 - 4\delta _1 \delta _2 (a+b)^2 >0 . \end{aligned}$$

#### Gierer–Meinhardt Reaction Kinetics

We also consider Gierer–Meinhardt reaction kinetics (Gierer and Meinhardt 1972). These have been used in a variety of theoretical and applied contexts and are particularly used as an example of Turing instabilities which lead to spatially localized patterns, such as spikes in 1-D domains and spots in 2-D domains (Wei and Winter 2013). We note in particular the study by Tse et al. (2010) of these kinetics on the sphere, demonstrating the effect of curvature on the stability and structure of patterns.

In non-dimensional form, these equations are:

47a $$\begin{aligned}&f(u,v) = 1+\frac{u^2}{v} -au , \end{aligned}$$

47b $$\begin{aligned}&g(u,v) = u^2 - bv, \end{aligned}$$

where the terms *au* and *bv* correspond to linear degradation of the morphogens, at rates *a* and *b*, respectively, and the terms involving $u^2$ correspond to the activator’s production of both *u* and *v*. We note the addition of the constant 1 to the activator kinetics, corresponding to basal production of the activator, which allows for robust spontaneous spot formation (Page et al. 2005; Krause et al. 2018b).

As for the Schnakenberg reaction kinetics, we find the homogeneous steady state of Eq. (47) by setting $f=0=g$. This gives the steady-state $\mathbf {u}^*$ and the Jacobian of the linearized system $\varvec{J}^*$:

48 $$\begin{aligned} \mathbf {u}^* = \begin{pmatrix} \dfrac{b+1}{a},&\dfrac{(b+1)^2}{ba^2} \end{pmatrix} , \qquad \varvec{J}^* = \begin{pmatrix} a\dfrac{b-1}{b+1} &{} -\left( \dfrac{ab}{(b+1)}\right) ^2 \\ \dfrac{2b+2}{a} &{} - b \end{pmatrix}. \end{aligned}$$

The conditions for Turing instability (3) are:

49a $$\begin{aligned}&a(1+(a-1)b^2) >0, \end{aligned}$$

49b $$\begin{aligned}&ab >0, \end{aligned}$$

49c $$\begin{aligned}&a(b^2-1)\delta _1-a^2b^2\delta _2 >0, \end{aligned}$$

49d $$\begin{aligned}&\left( a(b^2-1)\delta _1-a^2b^2\delta _2\right) ^2 - 4 \delta _1 \delta _2 ab >0. \end{aligned}$$

#### FitzHugh–Nagumo Reaction Kinetics

The final type of reaction scheme we consider are the FitzHugh–Nagumo kinetics, derived independently by FitzHugh (1955, 1961) and Nagumo et al. (1962) which is a two-component simplification of the famous Hodgkin–Huxley model (Hodgkin and Huxley 1952), which itself was proposed following experiments with the giant squid axon. The Hodgkin–Huxley equations model the potential across a surface membrane as the movement of potassium and sodium ions (plus a small leakage current of other ions). The FitzHugh–Nagumo reaction kinetics can lead to Turing–Hopf bifurcations (Castillo et al. 2016), as well as a multitude of behavior due to bistability (Keener and Sneyd 1998). These kinetics have also been used to model labyrinthine patterning in neurogenesis (Cartwright 2002). For simplicity, we only consider parameters where there is a unique positive steady state and we use parameters which are not in the Turing–Hopf bifurcation regime, in order to focus on the behavior of stationary patterns under growth. The homogeneous steady state in this case will only be unstable to modes arising from a pure Turing bifurcation. The FitzHugh–Nagumo kinetics can be written as

50a $$\begin{aligned}&f(u,v) = c \, \left( u - \frac{u^3}{3} +v - d \right) , \end{aligned}$$

50b $$\begin{aligned}&g(u,v) = - \left( \frac{u-a + bv}{c} \right) , \end{aligned}$$

where $a, \ b, \ c, \ d >0$ are constant parameters chosen to satisfy the Turing instability conditions given in Sect. 2.1, and in this case we take $\delta _1 =1$ and $d=0.6$. Following the analysis of Castillo et al. (2016), we require

51 $$\begin{aligned} 0< b < 1, \quad \text {and} \quad \left( -d + \frac{a}{b} \right) >0, \end{aligned}$$

to ensure that there is a unique positive steady state for the homogeneous problem. That is, there is only one positive solution to the system $f(u,v) = 0 = g(u,v)$, which is the intersection of the nullcline curves $v_1(u) = \frac{a-u}{b}$ and $v_2(u)= \frac{u^3}{3} - u + d$. This steady-state $\mathbf{u}^* = ( u^*, v^*)$ is given by

52a $$\begin{aligned} u^*&= \root 3 \of { \frac{3}{2} \bigg (-d+ \frac{a}{b} \bigg ) + \frac{1}{2} \sqrt{9 \bigg (-d + \frac{a}{b} \bigg )^2 + 4 \bigg (\frac{1}{b} -1 \bigg )^3} } \nonumber \\&\quad + \root 3 \of { \frac{3}{2} \bigg ( -d+ \frac{a}{b} \bigg ) - \frac{1}{2} \sqrt{9 \bigg (-d + \frac{a}{b} \bigg )^2 + 4 \bigg (\frac{1}{b} -1 \bigg )^3} }, \end{aligned}$$

52b $$\begin{aligned} v^*&= \frac{a}{b} - \frac{1}{b} \root 3 \of { \frac{3}{2} \bigg (-d+ \frac{a}{b} \bigg ) + \frac{1}{2} \sqrt{9 \bigg (-d + \frac{a}{b} \bigg )^2 + 4 \bigg (\frac{1}{b} -1 \bigg )^3} } \nonumber \\&\quad + \root 3 \of { \frac{3}{2} \bigg ( -d+ \frac{a}{b} \bigg ) - \frac{1}{2} \sqrt{9 \bigg (-d + \frac{a}{b} \bigg )^2 + 4 \bigg (\frac{1}{b} -1 \bigg )^3} }, \end{aligned}$$

noting that $v^* = \frac{a-u^*}{b}$ and thus that we require $0< u^* < a$ for $v^*> 0$, to obtain a positive steady state. Thus, the steady-state $\mathbf{u}^*$ and Jacobian ${\varvec{J}}^*$ are

53 $$\begin{aligned} \mathbf {u}^* = \begin{pmatrix} u^*,&\dfrac{a - u^*}{b} \end{pmatrix} , \qquad \varvec{J}^* = \begin{pmatrix} c \, \left( 1- {u^*}^2 \right) &{} c \, \\ -\dfrac{1}{c} &{} - \dfrac{b}{c} \, \, \end{pmatrix}. \end{aligned}$$

The Turing instability conditions, for the instability of the steady-state $\mathbf {u}^*$ are,

54a $$\begin{aligned}&c^2 \left( 1 - {u^*}^2 \right) - b >0, \end{aligned}$$

54b $$\begin{aligned}&1 - b \left( 1- {u^*}^2 \right) >0, \end{aligned}$$

54c $$\begin{aligned}&\delta _2 c^2 \left( 1 - {u^*}^2 \right) - b >0, \end{aligned}$$

54d $$\begin{aligned}&\left( \delta _2 c^2 \left( 1 - {u^*}^2 \right) - b \right) ^2 - 4 c^2 \delta _2 \left( 1 - b \left( 1- {u^*}^2 \right) \right) >0. \end{aligned}$$

### Numerical Approach

Throughout the rest of the paper, we will demonstrate solutions to equations (25) or (41) for different reaction kinetics *f* and *g*, as well as in different growth scenarios. We will use the commercially available finite element solver COMSOL, version 5.3, which will discretize the manifolds using second-order triangular finite elements. We note that these simulations were checked in various static domain cases using the Matlab package Chebfun (Townsend and Trefethen 2013; Townsend et al. 2016), in addition to convergence checks in spatial and time discretizations. In all simulations, we used a relative tolerance of $10^{-5}$ and fixed an initial time step of $10^{-6}$ (but let the solver increase the time step freely as the solution evolved). Convergence in time was checked by restricting the maximum time step, and convergence in space was determined via using different numbers of finite elements and comparing the norm of solutions over time and space. For the growing ellipse, we used 25,970 domain elements, and for the ellipsoid we used 19,704 boundary elements, which we now describe in some detail.

We note that one advantage of this choice of finite element software, as well as the restriction to dilational growth, is that it allows for simple implementations of growing manifolds where the growth is directed in particular directions in the ambient space. This is because the Laplace–Beltrami operator on a surface of dimension *n* can be constructed from the Laplace operator in the ambient space $\mathbb {R}^{n+1}$ (Dziuk and Elliott 2007, 2013; Olshanskii and Xu 2017), so that dilation of a particular coordinate in $\mathbb {R}^{n+1}$ allows a natural construction of the Laplace–Beltrami operator on the surface. We note that the planar ellipse model given by (25) is a flat 2-D manifold, and so the projection in this case is trivial. For Eq. (41), however, we can define both the Laplace–Beltrami operator and the dilution term in the ambient space $\mathbb {R}^3$ as the operators

55a $$\begin{aligned} \nabla ^2\equiv & {} \frac{1}{A(t)^2}\frac{\partial ^2 }{\partial x^2}+\frac{1}{B(t)^2}\frac{\partial ^2 }{\partial y^2}+\frac{1}{C(t)^2}\frac{\partial ^2 }{\partial z^2}, \end{aligned}$$

55b $$\begin{aligned} \nabla \cdot \mathbf {Q}\equiv & {} \frac{\left( \frac{ABz}{C}\right) ^2 \gamma _{AB}+\left( \frac{ACy}{B}\right) ^2 \gamma _{AC}+\left( \frac{BCx}{A}\right) ^2 \gamma _{BC}}{\left( \frac{ABz}{C}\right) ^2+\left( \frac{ACy}{B}\right) ^2 +\left( \frac{BCx}{A}\right) ^2}\nonumber \\= & {} \frac{A^4B^4z^2 \gamma _{AB}+A^4C^4y^2 \gamma _{AC}+B^4C^4x^2 \gamma _{BC}}{A^4B^4z^2+A^4C^4y^2+B^4C^4x^2}, \end{aligned}$$

with the $\gamma$’s defined by (40), and the variables (*x*, *y*, *z*) from the extrinsic coordinate system restricted to the surface. We can then define (41) in the whole space $\mathbb {R}^3$ using (55) and simply project the equations to the unit sphere in order to obtain the correct dynamics on a growing ellipsoid. We also note that this ambient formulation allows us to see an explicit interaction between curvature and anisotropic growth—namely, the dilution term (55b) contains explicit spatial heterogeneity due to curvature and anisotropy, as these do not appear in the planar ellipse, nor in the isotropically growing sphere described in Plaza et al. (2004).

### Robustness of Patterning to Growth

We now outline our systematic study of these three kinds of reaction kinetics on the elliptical and ellipsoidal domains, with dynamics governed by Eqs. (25) and (41), respectively. We vary the functional forms of *A*(*t*), *B*(*t*), and in the ellipsoidal case, *C*(*t*) in order to elucidate effects due to growth rates, anisotropy, and curvature. We consider different forms and rates of growth, in addition to the case of no growth. We note that on many domains, multistability of several non-uniform patterns is generic, especially in manifolds with dimension greater than one (Hunding 1980; Jensen et al. 1993; Borckmans et al. 1995). Because of this, we will emphasize qualitative differences between the kinds of patterns, rather than quantitative differences in the final patterned state. We now describe all of the different growth scenarios, which correspond to different choices in the parameters, manifolds, growth functions, etc. We will always take parameters within the Turing regime, so that the homogeneous steady state is unstable and leads to the formation of stationary patterns. Unless otherwise mentioned, we will always assume initial data of the form $\mathbf {u}(0) = \mathbf {u}^* + \xi$, where $\mathbf {u}^*$ is the spatially homogeneous steady state and $\xi$ is a normally distributed spatial random variable with zero mean and standard deviation $10^{-3}$. We note that the same realization of the spatial noise was used in all simulations.

For each scenario, we will consider a fixed static domain $\varOmega ^*$, such as a sphere or an ellipsoid, and compare it to a growing domain $\varOmega (t)$ such that after a time period *T*, $\varOmega (T) = \varOmega ^*$. We are interested in the equilibrium states on the final manifold, and we are also interested in the evolution of non-uniform states. For these reasons, for each scenario we identify a kinetic timescale $T^*$ such that the reaction–diffusion equations have approximately reached their steady-state behavior. We determine $T^*$ by simulating the equations on the static manifold $\varOmega ^*$ and observe when the patterning process settles into a stationary state, and we check that no further pattern movement or creation occurs in the time period up to $10T^*$. With such a kinetic timescale in hand, we always let the pattern develop fully before initiating growth, and we always let the pattern equilibrate after growth. Mathematically this means that we take $\varOmega (t)=\varOmega (0)$ for $t \le T^*$ and $\varOmega (t)=\varOmega (T)$ for $t \ge T-T^*$, so that growth is confined to the interval of time $t \in (T^*, T-T^*)$. We will always begin in a symmetric manifold, so that $\varOmega (0)$ is either a circular disk or a sphere, and we will take the initial radius $r_0$ to be large enough so that patterning occurs.

We use the following functions to characterize the type of growth used and state these in terms of growth of the axis *A*(*t*) and assume that this axis has an initial value of $A(0)=r_0$ and a final value of $A(T)=r_T$. Note that these functional forms are applied analogously to all growing axes. We also note that these functions are constant at the beginning and end of the simulation time, as described above. We define linear growth, where the domain is growing at a constant rate, by:

56 $$\begin{aligned} A(t) = r_0 + \left( \frac{r_{T} - r_0}{T-T^*} \right) (t-T^*) , \quad t \in (T^*, T-T^*). \end{aligned}$$

Linear growth is unlikely to occur in biological circumstances (Plaza et al. 2004); however, it is a simple form of growth frequently used in the literature and so worth investigating. We consider sublinear growth, where the domain is growing at an increasing rate (i.e., $\ddot{A}(t) > 0$), given by:

57 $$\begin{aligned} A(t)= r_0 \exp \left( \log \left( \frac{r_{T}}{r_0} \right) \frac{t-T^*}{T-T^*} \right) , \quad t \in (T^*, T-T^*). \end{aligned}$$

This growth function is exponential, which is a reasonable model for certain biological growths—e.g., for the initial growth phases of some tissues during development. Superlinear growth, where the domain is growing at a decreasing rate (i.e., $\ddot{A}(t) < 0$), is defined by:

58 $$\begin{aligned} A(t)=r_0 + (r_{T} - r_0) \left( 1 - \exp \left( -\frac{5}{T-T^*} (t-T^*) \right) \right) . \end{aligned}$$

This is a logistic-like growth, which is a realistic approximation of growth for some biological applications, where growth is reliant on limited external resources which can become scarce as they are depleted. We also use a punctuated form of growth defined by alternating piecewise constant and linear regions during the growing phase, but do not explicitly give it for brevity (as we will see, the particular growth function plays almost no role in the qualitative structure of the steady states). To ensure the numerical solver did not encounter any problems due to lack of continuity at $t= T_f$, the growth function is smoothed to be continuous with a transition region of 0.01 at $t=T^*$ and $t=T-T^*$.

We consider two different growth scenarios: staged growth with a symmetric final manifold, or pure dilational growth with an elliptical or ellipsoidal final manifold. In the former, *A*(*t*) and *B*(*t*) (and in the ellipsoidal case *C*(*t*)) grow in two (three) different stages subsequently and reach the same final value of $r_T$, so that the final domain is a larger disk (sphere). We note in this case that we also considered an analogous isotropic growth scenario for the same manifold (e.g., without stages). In the second scenario, we consider a final manifold which is not symmetric, and so only one of the axes grows leading to an elongated ellipse or ellipsoid at the final time $t=T$. Additionally, we consider the effect of the timescale over which the domain grows on the patterns formed, denoted as $T_g = T-2T^*$. We consider three different timescales: fast growth given by $T_g = T^*/2$, growth comparable to the kinetic timescale so $T_g = T^*$, and growth which is much slower given by $T_g = 10T^*$. We note that $T^*$ is somewhat arbitrary, and in our simulations we take it to be sufficiently large to ensure we are in a patterned state, but we believe these growth timescales at least highlight differences in how the rate of growth influences the evolution of patterns. We note that in staged growth, we increase the growth timescale so that growth in each direction occurs over the same period of time $T_g$.

**Table 1:** List of the various parameter values studied in our systematic study

Name	Kinetics	Parameters	$T^*$	Static behavior
S1	Schnakenberg	$a=0.05, b=1.5, \delta _2=100$	5000	Spots
S2	Schnakenberg	$a=0.05, b=1.6, \delta _2=20$	60,000	Labyrinthine
GM1	Gierer–Meinhardt	$a=0.8, b=5.5, \delta _2=100$	13,000	Large spots
GM2	Gierer–Meinhardt	$a=1.5, b=3, \delta _2=300$	13,000	Small spots
FHN1	FitzHugh–Nagumo	$a=0.8, b=0.4, \delta _2=100$	2000	Spots
FHN2	FitzHugh–Nagumo	$a=0.6, b=0.99, \delta _2=50$	8000	Labyrinthine

In all cases, we took $\delta _1=1$ and for FitzHugh–Nagumo kinetics took $c=1$ and $d=0.6$. For Schnakenberg and Gierer–Meinhardt on both domains, and FitzHugh–Nagumo on the ellipsoidal domain, the initial manifold was always taken with $r_0=10$ and the growth was to a maximum of $r_T=4r_0$, whereas for FitzHugh–Nagumo on the planar ellipse we used $r_0=25$ and the growth to a maximum of $r_T=4r_0$

We ran simulations of Eqs. (25) and (41) for each of the three reaction kinetics described in Sect. 3.1. In general, each of these models can give rise to a wide variety of behaviors, but for simplicity we consider the 6 parameter sets shown in Table 1. In each case, we explored all combinations of the 4 growth functions, 3 timescales, and both the final manifold scenarios (including an isotropic growth case), as well as 12 simulations on the static domain given by $\varOmega ^*=\varOmega (T)$, leading to a total of 444 simulations. In each case, we compared the final pattern (in terms of the activator, *u*, as the inhibitor can be related to it via a phase), and we summarize differences below. We emphasize that many simulations will lead to quantitatively different steady states (e.g., the number of spots may differ slightly), but these quantitative differences will also occur due to different realizations of the initial data, as the precise organization of the final non-uniform state can sensitively depend on the initial data (Maini et al. 2012). Additionally, we ran other simulations with variations in these parameters or growth scenarios to ensure that our results were robust. In particular, we did not systematically vary staged growth at different rates, but did explore some simulations in this case to understand the possible behaviors.

Broadly speaking, we found that for Schnakenberg and Gierer–Meinhardt kinetics, the kinds of patterns observed and their qualitative properties did not change much, except due to the rate of growth, or whether or not the final domain was curved. The functional form of growth is particularly unimportant, only really changing the ‘effective’ rate of growth in some boundary cases which we will describe below. This leads us to conjecture that for the long-time steady states observed, linear growth with varying rates in growing domains is sufficient to capture all of the qualitative dynamics that can be observed, and that static domain simulations capture many qualitative properties of the final patterned state for these kinds of reaction kinetics. FitzHugh–Nagumo kinetics result in a richer structure, in terms of its interaction with growth, curvature, and anisotropy in the domain or growth. We do note, however, that analogous comments about the functional form of growth can be made. As the functional form of growth only influences the rate of growth, we will only show figures using the case of linear growth, but remark that the results with all other growth laws were qualitatively identical throughout. Similarly, we only show cases where noticeable effects occurred, and omit figures with comparable behavior to those shown. Finally, we note that solutions using the Schnakenberg and Gierer–Meinhardt kinetics will always be positive, as these models correspond to morphogen concentrations, whereas the activator *u* in FitzHugh–Nagumo is the voltage with respect to an arbitrary gauge and hence can locally take positive and negative values.

In the following sections, we shall employ this systematic approach to study Turing patterns on growing domains in the case where the domain is a flat ellipse in Sect. 4 and in the case where the domain is an ellipsoid surface in Sect. 5. We then turn our attention to the study of striped patterns on both surfaces, in Sect. 6.

**Fig. 1:**
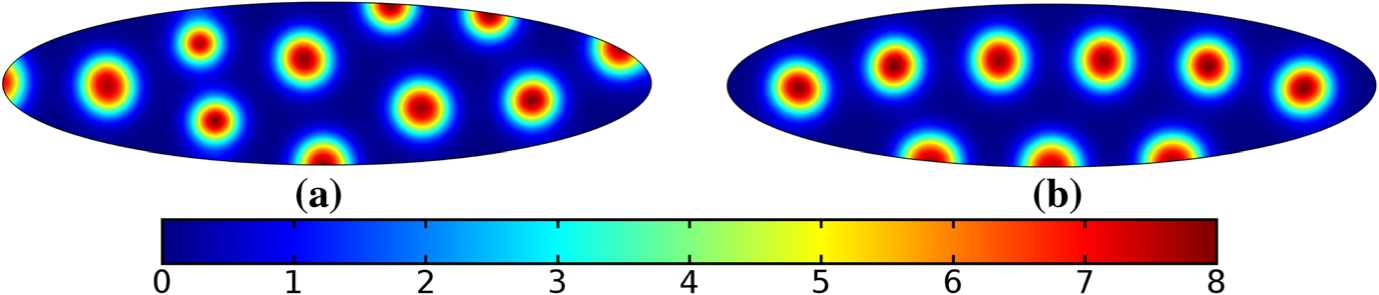
Simulations of Eq. (25) with kinetics given by (44) using parameters S1 for growth only along the *x*-axis. We plot the final distribution of *u* on **a** the static domain and **b** a domain with linear growth over the timescale $T_g=10T^*=50{,}000$ (Color figure online)

**Fig. 2:**
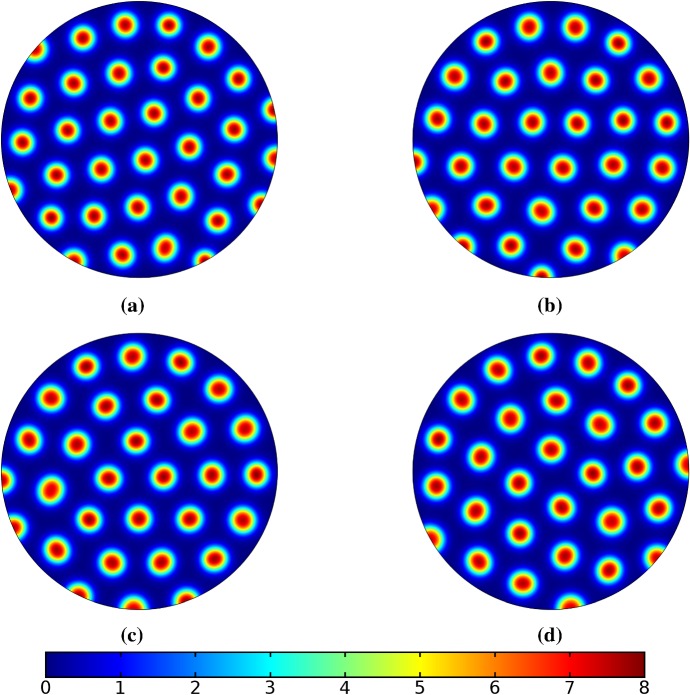
Simulations of Eq. (25) with kinetics given by (44) using parameters S1. We plot the final distribution of *u* on **a** the static domain, **b** a domain with linear isotropic growth over the timescale $T_g=T^*/2=2500$, **c** a domain with linear isotropic growth over the timescale $T_g=10T^*=50{,}000$, and **d** staged growth over the timescale $T_g + T_g = 2 \times 10T^* = 100{,}000$ (Color figure online)

## Reaction–Diffusion Systems on Planar Elliptical Domains

We now describe some of the results obtained in the case of Schnakenberg and Gierer–Meinhardt kinetics in the case of a planar elliptical domain. In Fig. 1, we compare a static ellipse with an ellipse having grown from an initial circle with growth only along the semimajor axis of the final ellipse. We note that growth appears to generate a more symmetric final distribution of spots (and that this result is identical across growth rates and functions in this case). This is consistent with the robustness attributed to growth in 1-D domains (Crampin et al. 1999). We also consider isotropic and staged growth for a circle in Fig. 2, here showing that isotropic growth, staged anisotropic growth, and the static domain all admit a distribution of spots with comparable symmetry. We remark that while some mild robustness under growth is generally independent of growth rates, it is also a small effect that depends on the initial data and the nature of the domain itself, and is in a sense only a weak qualitative effect. Overall, we find that formation of spot patterns in Schnakenberg is qualitatively independent of growth altogether, at least in two spatial dimensions. For all further plots, we omit any simulation which is qualitatively identical to one already presented.

**Fig. 3:**
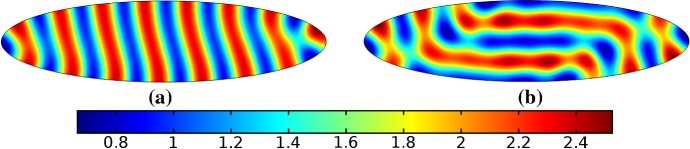
Simulations of Eq. (25) with kinetics given by (44) using parameters S2 for growth only along the *x*-axis. We plot the final distribution of *u* on **a** the static domain and **b** a domain with linear growth over the timescale $T_g=10T^*=50{,}000$ (Color figure online)

**Fig. 4:**
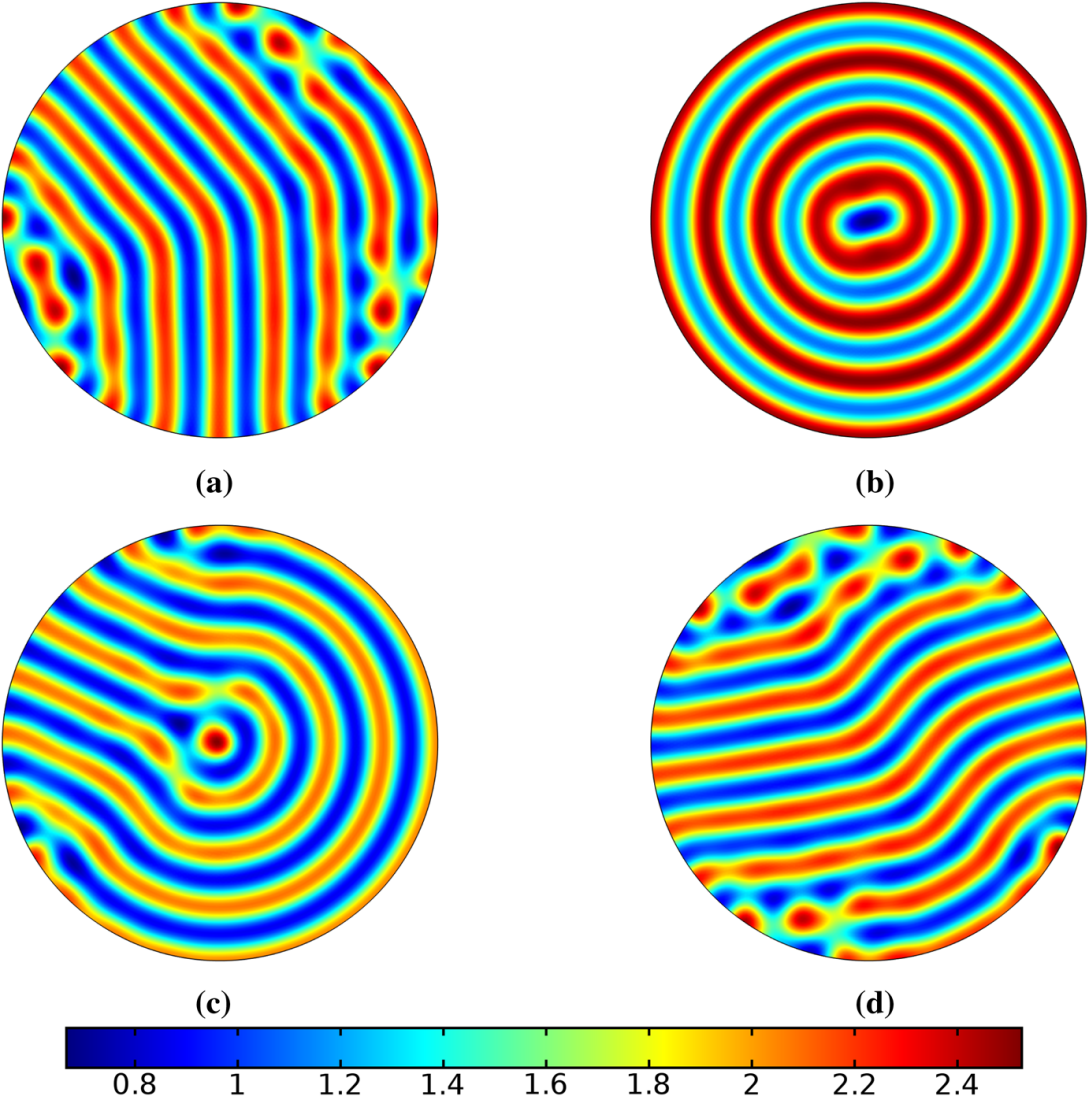
Simulations of Eq. (25) with kinetics given by (44) using parameters S2. We plot the final distribution of *u* on **a** the static domain, **b** a domain with linear isotropic growth over the timescale $T_g=T^*/2=2500$, **c** a domain with linear isotropic growth over the timescale $T_g=10T^*=50{,}000$, and **d** staged growth over the timescale $T_g + T_g = 2 \times 10T^* = 100{,}000$ (Color figure online)

We give analogous plots of the elliptical and circular domains in Figs. 3 and 4, respectively. Similar to the case of spot patterns, the patterns observed in the ellipse in Fig. 3 were essentially insensitive to growth of any rate or functional form, but unlike spots, these patterns were qualitatively different to simulations on a static domain. In particular, we suspect that the thin static ellipse gives a proclivity to vertically symmetric stripes forming, whereas growth at any rate will distort this structure. As before, the functional form of growth did not matter much in any case, but the growth rate and level of isotropy played a much larger role in the circular domain, shown in Fig. 4. Here, isotropic growth which was fast (Fig. 4b) gave rise to symmetrical patterns. Slow isotropic growth (Fig. 4c) gave more circularly symmetric patterning than in the static domain (Fig. 4a), but we note that this is not perfectly circular patterning, so that there is still some sensitivity to the initial data. Finally, anisotropic (staged) growth (Fig. 4d) gave rise to patterns indistinguishable from simulations on a static domain; note in particular that here the pattern is almost a rotation of that shown in Fig. 4a.

**Fig. 5:**
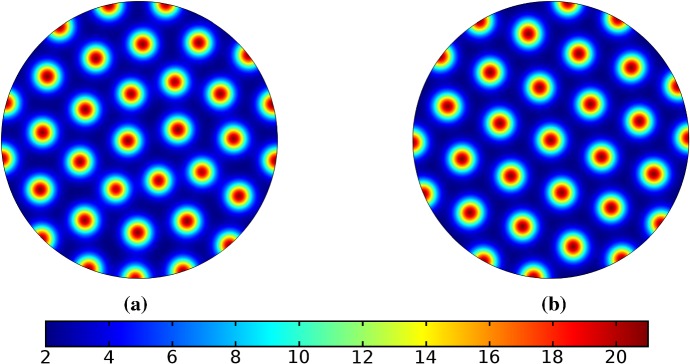
Simulations of Eq. (25) with kinetics given by (47) using parameters GM1. We plot the final distribution of *u* on **a** the static domain and **b** a domain with linear isotropic growth over the timescale $T_g=10T^*=130{,}000$ (Color figure online)

Turning now to simulations of the Gierer–Meinhardt kinetics (47), we plot some example simulations in Fig. 5. As shown in Fig. 2, the case of slow isotropic growth gives rise to some symmetry to the final patterned state, but this was not observed for either staged growth or faster growth regimes, which gave the final spot patterns comparably disordered to the static growth case. We note that in the case of the final domain being elliptical, all growth scenarios gave rise to comparably disordered final patterns. We note the inclusion of the constant term (feed rate) in Eq. (47) leads to generally robust dynamics. If this term was not present, then after the initial production of spots, changes to the manifold do not lead to spot splitting or production of new spots. Growth in the case without this feed rate will, in all cases we explored, lead to transport of spot solutions, but no meaningful interactions or new behaviors.

**Fig. 6:**
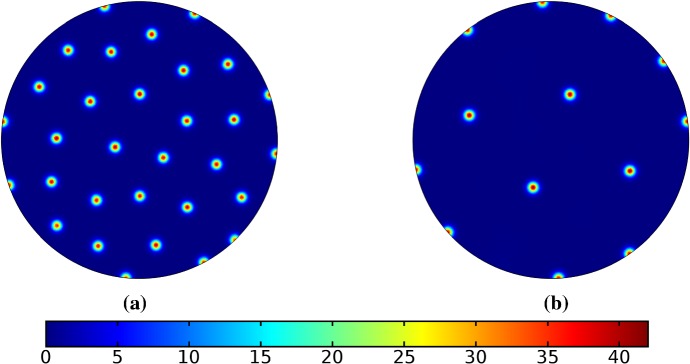
Simulations of Eq. (25) with kinetics given by (47) using parameters GM2. We plot the final distribution of *u* on **a** the static domain and **b** a domain with linear isotropic growth over the timescale $T_g=10T^*=130{,}000$ (Color figure online)

We also consider Gierer–Meinhardt simulations in the case of small isolated spot solutions (parameter set **GM2** from Table  1) and plot an example in Fig. 6. We note that the static domain gives rise to many more spots than the case of isotropic growth. In fact, staged growth or isotropic growth at all rates exhibits the same number of spots in analogous configurations as shown in the case of isotropic growth. We suspect this is due to a conservation of spots inherent in the system, as discussed above, despite a positive feed rate. This occurs here as the inhibitor is able to suppress the constant production of activator throughout the domain (due to a much larger diffusion rate of the inhibitor and a smaller degradation rate). New spots are formed as the domain grows, but typically on the boundary and far from existing spots (which generate large regions of inhibition), so that the final number of spots in all growth cases is 12–14, compared with the 31 present in the static domain. This quantitative difference was consistent across 5 different realizations of the initial conditions (with different numbers of spots forming, but always less than 50% in the case of any growth). A similar effect was also observed in the elliptical domain (not shown).

**Fig. 7:**
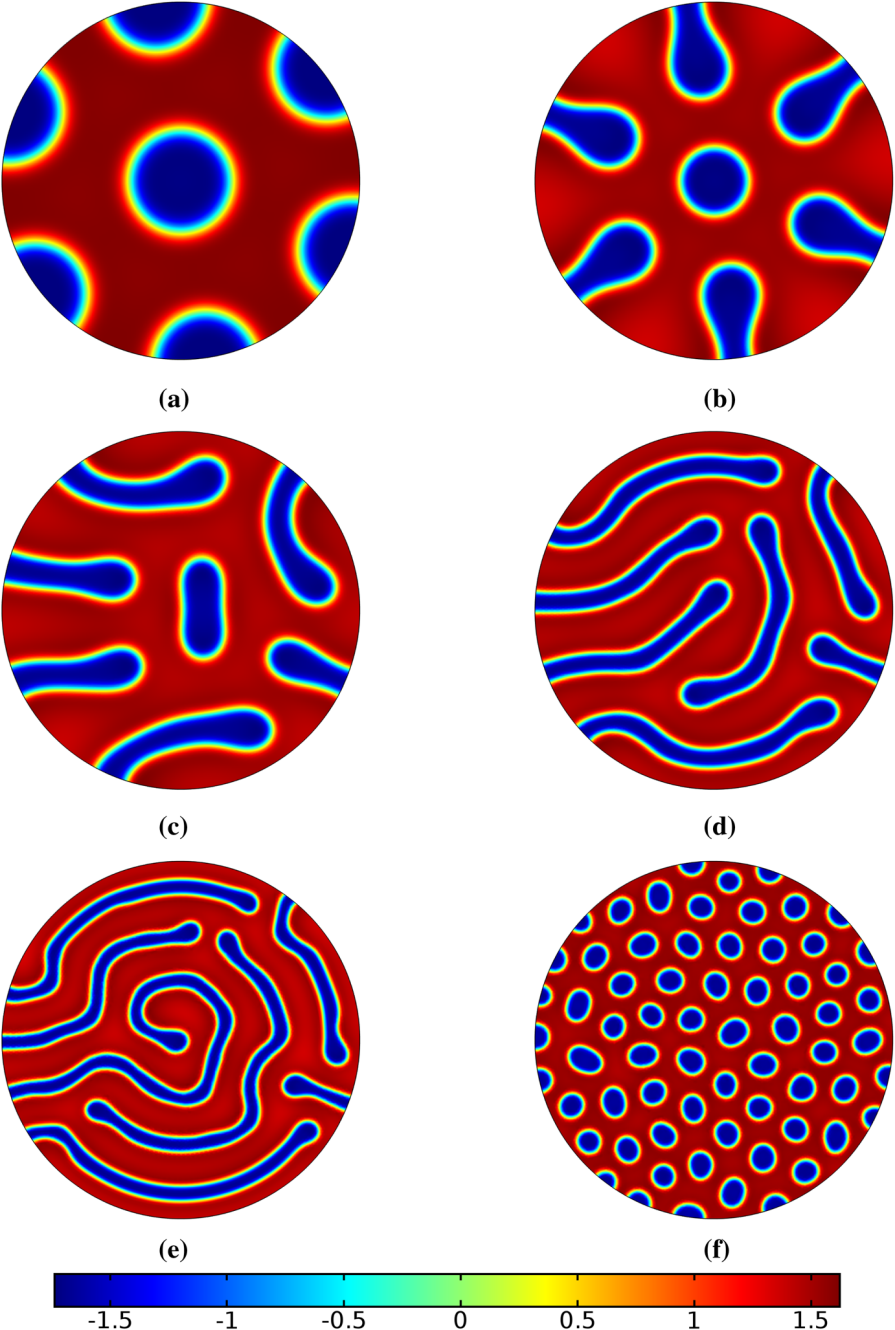
Simulations of Eq. (25) with kinetics given by (50) using parameters FHN1 for isotropic growth on a circular domain. We plot several distributions of *u* on a domain with linear isotropic growth over the timescale $T_g=10T^*=20{,}000$, at times **a**$T^*$ (fully developed pattern before growth), **b**$3T^*$, **c**$4T^*$, **d**$7T^*$, **e**$T=12T^*$ (the final distribution), and **f** the same simulation on a static domain. Also note that the domains are not shown to scale; **a** has a radius that is 4 times smaller than in **e** or **f** (Color figure online)

**Fig. 8:**
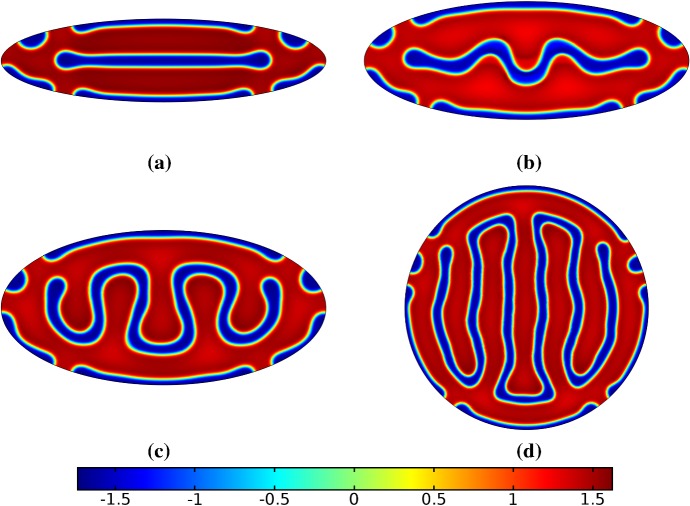
Simulations of Eq. (25) with kinetics given by (50) using parameters FHN1 for staged growth first in the *x*-axis only then in the *y* axis only. Note that at time $T^*$, the solution is identical to Fig. 7a. We plot several distributions of *u* on a domain with linear growth over the growth timescales of $T_g=10T^*=20{,}000$ in each direction. These are shown at times **a**$11T^*$ (fully grown in the *x*-direction), **b**$12.5T^*$, **c**$14T^*$, and **d** the final patterned state (Color figure online)

**Fig. 9:**
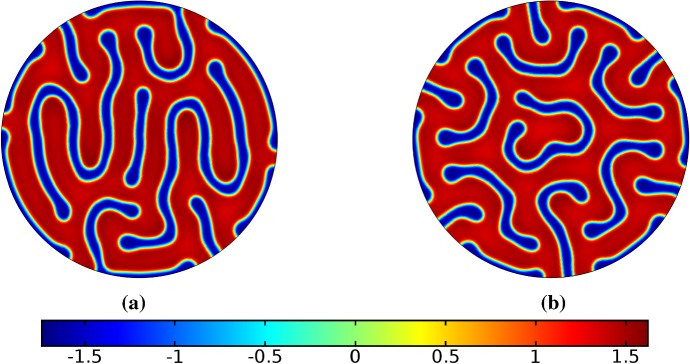
Simulations of Eq. (25) with kinetics given by (50) using parameters FHN1 for both staged growth and isotropic growth in a fast growth regime. Note that at time $T^*$, the solution is identical to Fig. 7a. We plot several distributions of *u* on a domain with linear growth over the growth timescales of $T_g=T^*/2=1000$ in each direction (**a**) and isotropically (**b**) (Color figure online)

Finally, we consider the FitzHugh–Nagumo kinetics given by (50) on the elliptical domain. For these kinetics, we observe substantial differences due to the growth of the domain, and these depend heavily on the kind of growth and marginally on the rate of growth; as in all other cases, the functional form of the time-dependent growth was equivalent to just using linear growth at a different rate. In Figs. 7 and 8, we show the evolution under isotropic and anisotropic growth of spot solutions to these equations. In both cases, after $T^*=2000$ units of time, the solutions are identical as shown in Fig. 7a. We note that in both cases, the number of minimal (blue) regions before growth and the number after is the same (7), but that the spots in the static domain are stretched and curved in complex ways, highly dependent on the mechanism of growth, as shown. Compared to the static circular domain in Fig. 7f, this is a strikingly different kind of patterning. We also note that the final patterned state here is stationary and robust against small numerical perturbations. We note that different initial conditions can tend to different long-time distributions, but these have comparable structures (e.g., in the staged growth case we always observe a central curved blue region). Finally, we remark that this conservation of minimal regions is only true for sufficiently slow growth, as the case of isotropic and staged growth shown in Fig. 9 demonstrates. We do note that there is still some qualitative difference between the kinds of growth in this case (namely, the staged growth has patterns more clearly oriented in the *y* direction), but the effect is weaker due to the introduction of more minimal (blue) regions.

**Fig. 10:**
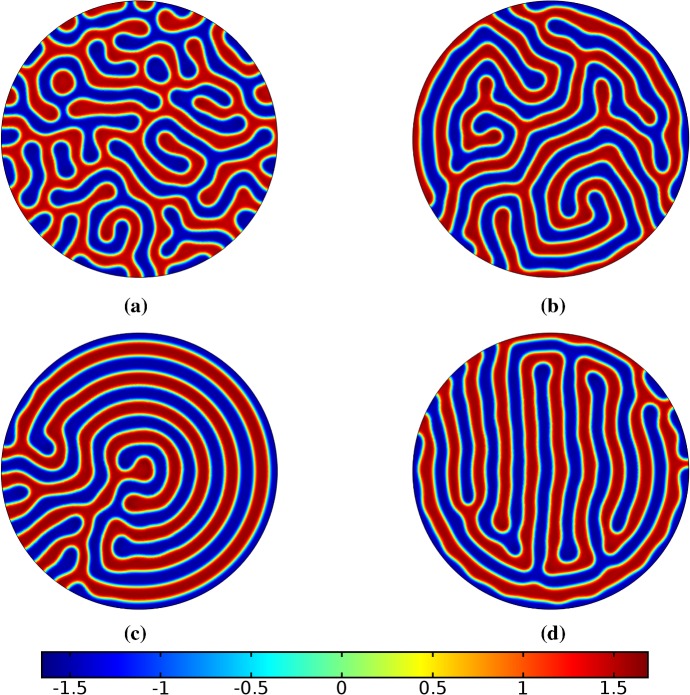
Simulations of Eq. (25) with kinetics given by (50) using parameters FHN2 for various kinds of growth. We plot several distributions of *u* on a domain with linear growth over various growth timescales. These are the **a** static domain, **b** isotropic growth over $T_g=0.5T^*$, **c** isotropic growth over $T_g=10T^*$, and **d** staged growth over $T_g=10T^*$ first in the *x* direction and then in the *y* direction (Color figure online)

**Fig. 11:**
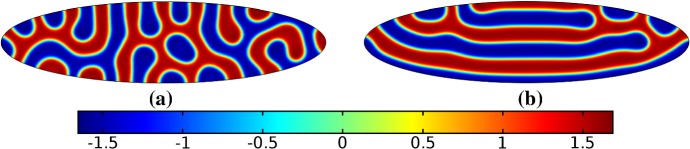
Simulations of Eq. (25) with kinetics given by (50) using parameters FHN2. In **a**, we show values of *u* on a static ellipse and in **b** over a growth timescale of $T_g=10T^*$ (Color figure online)

Lastly, we consider the evolution of labyrinthine solutions under different growth regimes for the FitzHugh–Nagumo kinetics. In Figs. 10 and 11, we give examples of this on the circular domain and the elliptical domain, respectively. As in the spot simulations, it is clear that growth can change the qualitative properties of the patterns, and that this can depend on both if the growth is isotropic or anisotropic. In all cases, we observe labyrinthine-like patterns, but see that slow isotropic growth (Fig. 10c) can induce circular striping in these patterns, and that slow staged growth (Fig. 10d) can also introduce alignment along the most recent growth direction (the *y* axis). All rates of growth changed the final elliptical state in qualitatively similar ways as shown in Fig. 11, in that both maximal and minimal regions align along the semimajor axis of the ellipse, which is also the only axis of growth.

**Fig. 12:**
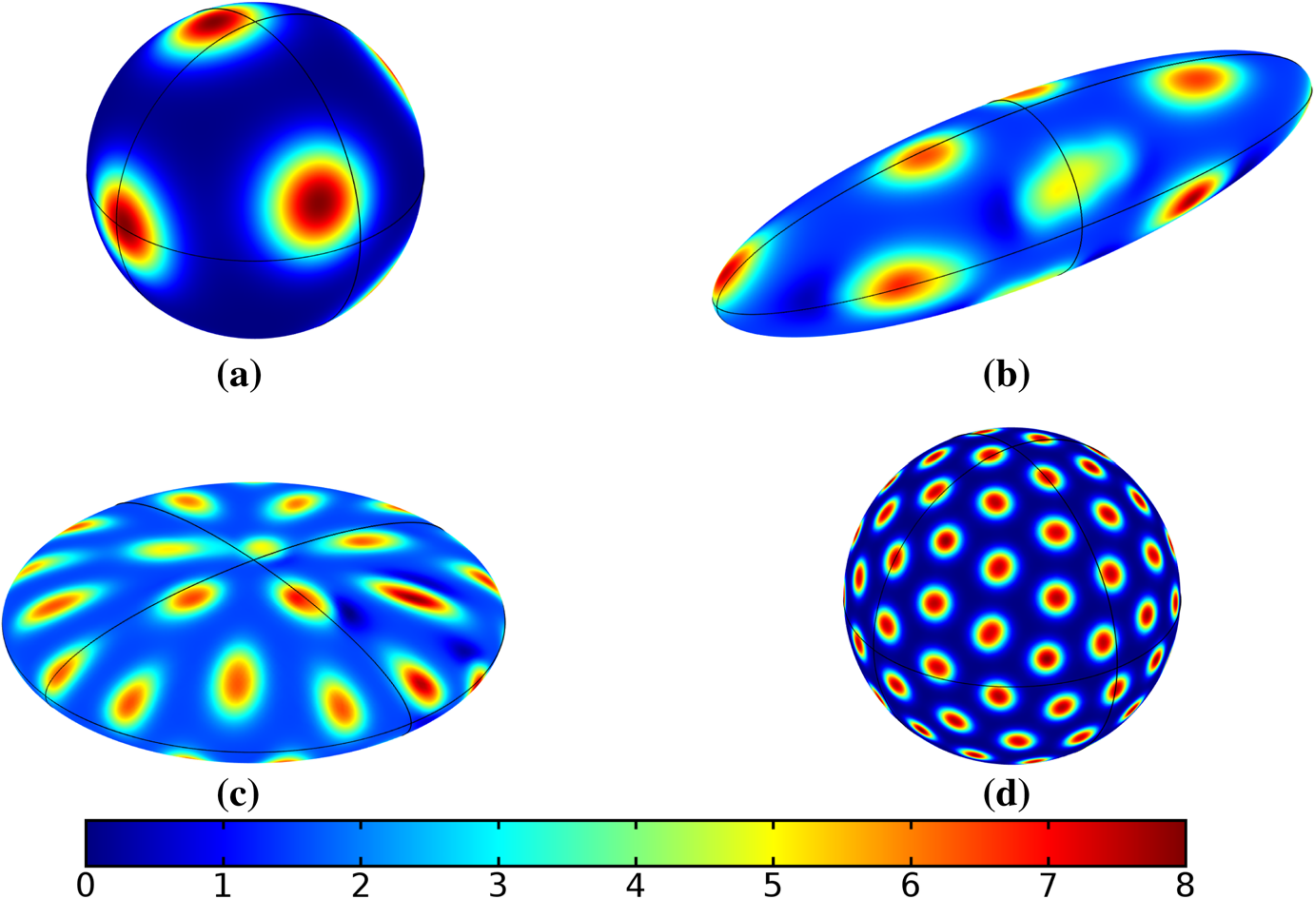
Simulations of Eq. (41) with kinetics given by (44) using parameters S1 over a growth timescale of $T_g=10T^*$. We plot the distribution of *u* on **a** the domain before growth, **b** the domain after growth along the *x* axis ($T^*+T_g$), **c** the domain after growth along the *x* and *y* axes ($T^*+2T_g$), and **d** the distribution of *u* on the final manifold (Color figure online)

## Reaction–Diffusion Systems on Ellipsoidal Domains

We now demonstrate solutions to Eq. (41) for the various reaction kinetics and growth regimes discussed. We note that now with three axes of possible growth, that there are three final possible domains (an oblate and a prolate spheroid or ellipsoid) where two axes are of equal size, depending on if one axis is smaller or larger than the other two. We did not systematically explore all intermediate domains with all variations in growth or kinetics, but we did include some in order to determine the influence of anisotropic curvature (as opposed to the isotropic curvature of a perfect sphere) on the final pattern. These effects can be seen during the transient staged growth, despite the shown states not being completely stationary, so we omit these results for brevity. Similarly, in all cases the effects of growth on different kinetics persist in these simulations, so we only show simulations where the influence of curvature (as well as new directions of anisotropic growth) can affect the final pattern. In particular, we do not include simulations of Gierer–Meinhardt, although we did observe the same kinds of patterns as shown in Figs. 5 and 6.

We begin by demonstrating the influence of curvature on Schnakenberg spot solutions in Fig. 12 for slow staged growth, which are analogous to those in Fig. 1. We see that the intermediate domains have spots which are not perfectly circular; as mentioned before, the patterns do not change much if the growth is stopped and the pattern is allowed to settle into a stationary solution on either of these non-spherical domains. Despite the manifold being locally planar (by definition of a two-dimensional manifold), the spots are large enough to be influenced by local curvature and anisotropy so that they do not resemble their planar analogues. Despite growth, however, the final patterned state on the larger sphere is qualitatively the same on static or grown manifolds, independent of any details of the growth, as in the planar case shown in Fig. 1.

**Fig. 13:**
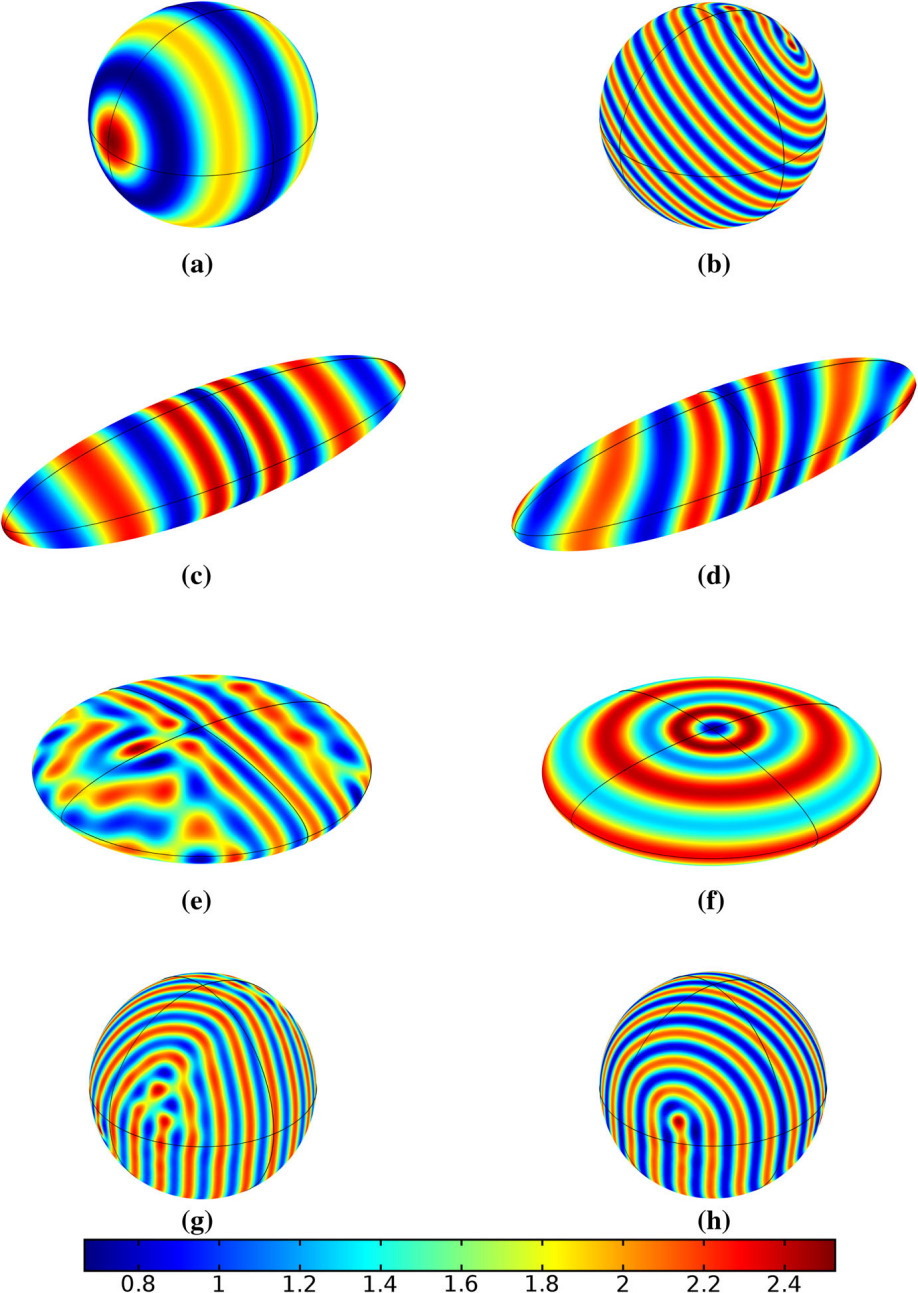
Simulations of Eq. (41) with kinetics given by (44) using parameters S2. We plot the distribution of *u* on **a** the domain before growth, **b** the final static domain, and in **c**–**h** key time points in staged growth for two different growth rates. Specifically, **c**, **e**, **g** use $T_g=10T^*$ and **d**, **f**, **h** use $T_g=T^*/2$, where **c**, **d** are at time $T^*+T_g$, **e**, **f** are at time $T^*+2T_g$, and **g**, **h** are at time $T^*+3T_g$ (Color figure online)

We next consider the evolution of labyrinthine Schnakenberg patterns in Fig. 13. Here we compare staged growth of these patterns in fast and slow regimes. The transient behaviors in the fast and slow growth regimes are quite different, but the final steady-state patterns are comparable with the fast growth case exhibiting some minor defects in the observed final labyrinthine pattern. We also note that the final patterns in the static domain and the slow growth domain, as shown in Fig. 13b, h, respectively, are essentially rotations of one another. Similar behavior is observed in the isotropic growth cases, where the final steady state differs from the static domain in the same way that different random initial data would (not shown).

**Fig. 14:**
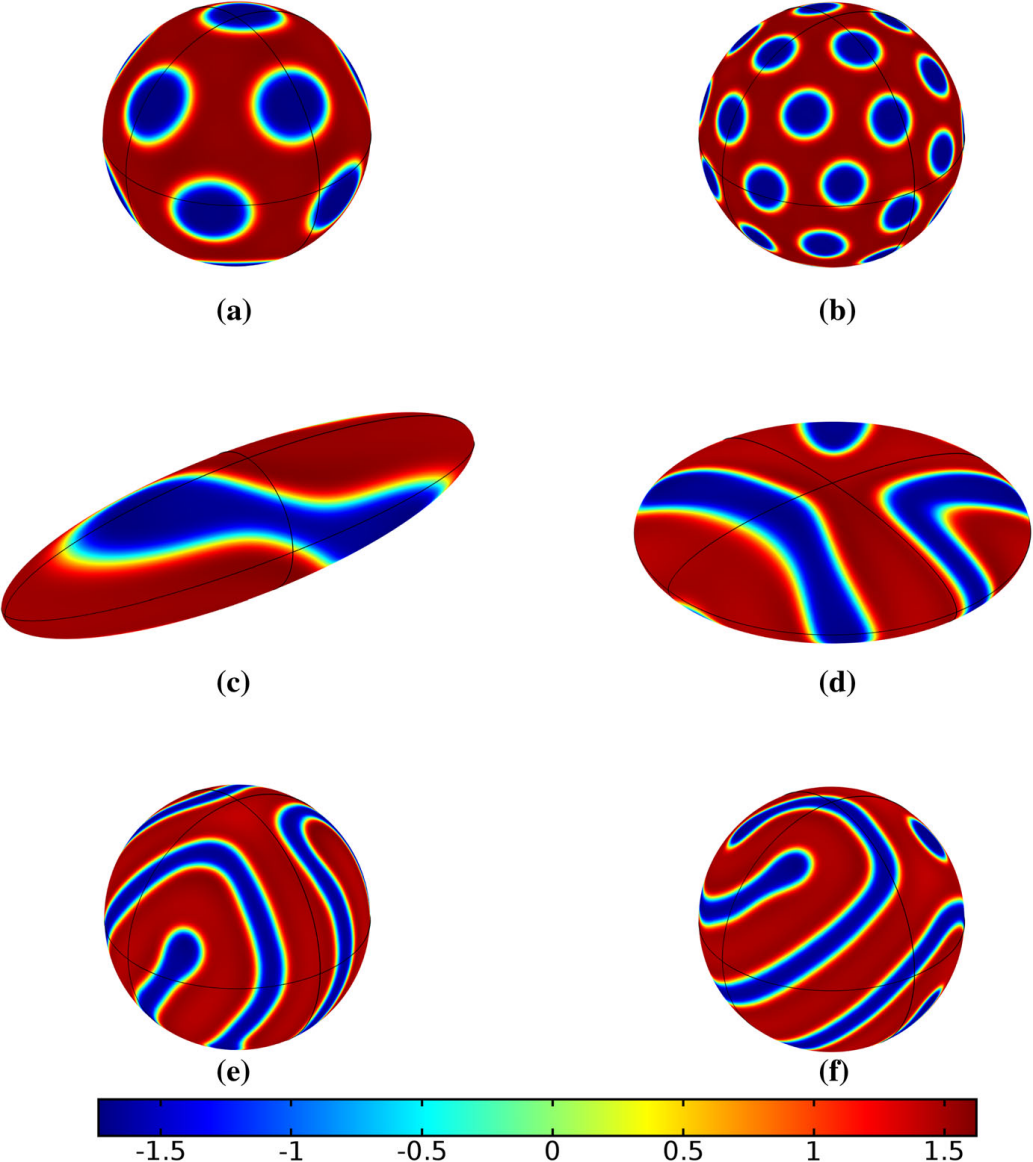
Simulations of Eq. (41) with kinetics given by (50) using parameters FHN1. We plot the distribution of *u* on **a** the domain before growth, **b** the final static domain, and grow the domain using staged growth over the timescale $T_g=10T^*$ in **c**–**e** where **c** is at $T^*+T_g$, **d** is at $T^*+2T_g$, **e** is the final stationary pattern in the staged growth case, and **f** is the isotropic growth case over the same growth period as in each staged direction (Color figure online)

We now consider the evolution of spot solutions using FitzHugh–Nagumo kinetics, which we show in Fig. 14. We note comparable behavior to the planar case shown in Figs. 7 and 9. Initially, three spots form on the domain of radius $r_0$, whereas the static domain with radius $4r_0$ has many more spots. Slow growth from the initial sphere, however, preserves these regions topologically (e.g., no new regions are formed), but they are twisted into curved patterns, which depend heavily on the directionality of growth and the domain. As in the planar case, growth which is sufficiently fast can induce the creation of new regions, leading to four minimal regions in staged growth over the growth timescale $T_g=T^*/2$ (for each direction) and eight minimal regions in isotropic growth over the same growth period. We recall that the staged growth occurs in each direction for a period of $T_g$ time units, so it is reasonable for fast isotropic growth in this case to produce more splitting, as more surface area is created in a shorter period of time. We note, however, that there is some sensitivity to exact geometries and growth rates that are sufficiently fast, so we do not pursue any further quantitative claims here.

**Fig. 15:**
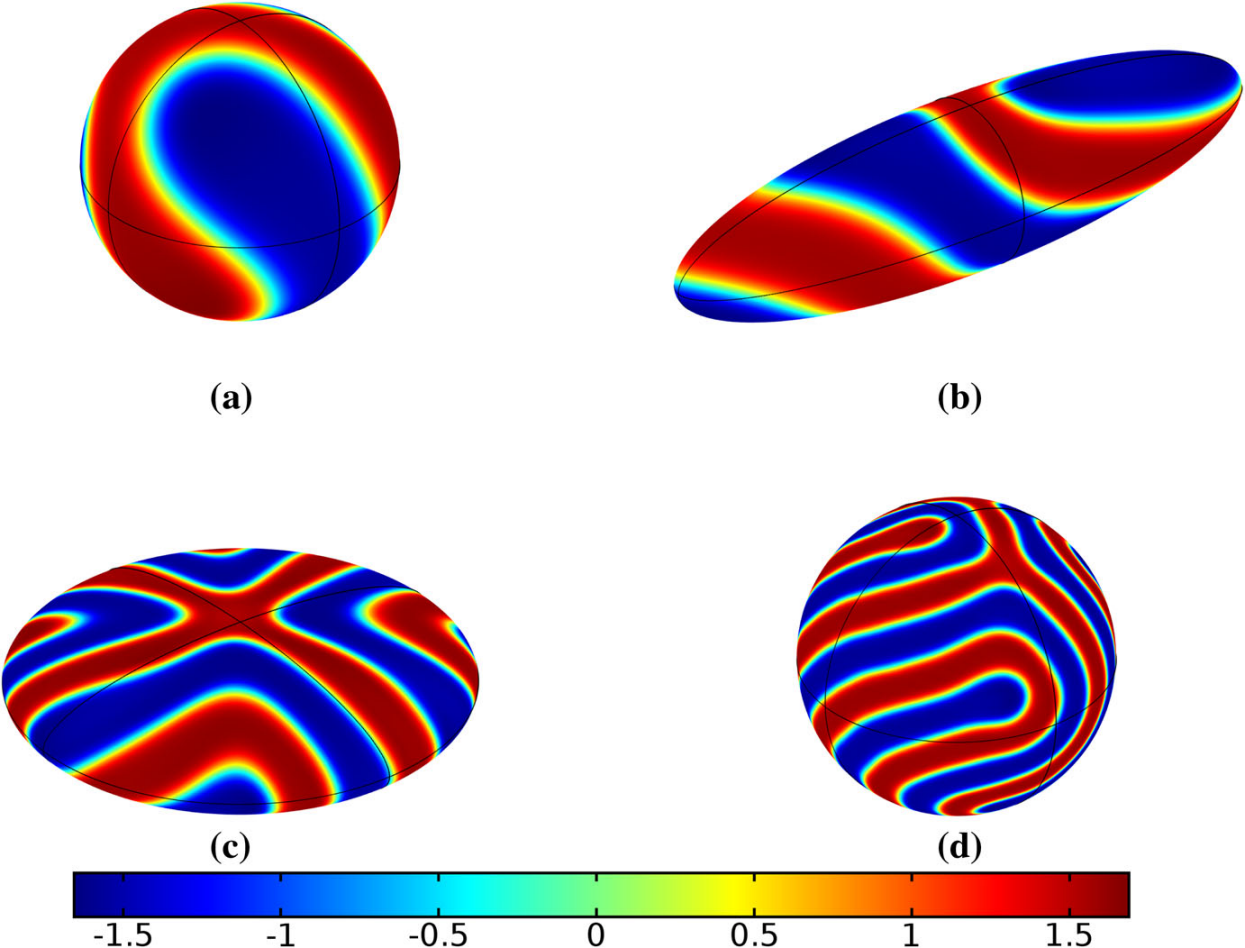
Simulations of Eq. (41) with kinetics given by (50) using parameters FHN2. We plot the distribution of *u* and grow the domain using staged growth over the timescale $T_g=10T^*$ where **a** is before growth at time $T^*$, **b** at time $T^*+T_g$, **c** at time $T^*+2T_g$, and **d** is the final stationary pattern (Color figure online)

Finally, we again consider the evolution of labyrinthine patterns under growth in Fig. 15. We again observe interesting patterns on the intermediate domains, but the effect of anisotropic growth is no longer obvious, as it was in the planar case shown in Figs. 10, 11 and 12. We also note that, independent of the growth rate or the use of static domains or isotropic growth, these figures are indicative of the kinds of patterns found in this case, and these variations do not seem to influence the qualitative behaviors. In all cases, a similar pattern emerges that appears qualitatively as one might expect when studying labyrinthine patterns on curved geometries, and growth only (qualitatively) seems to change one pattern into another without any stark differences due to the growth itself.

## Stripe Stability in Isotropic and Anisotropic Growth Regimes

Having systematically explored a wide range of reaction kinetics and growth regimes on planar and curved domains, we now briefly mention some results concerning the numerical stability and evolution of target and stripe patterns under growth. These patterns are special solutions that typically require spatial heterogeneity or specific initial data in order to observe (although we note that growth can do this as well, as shown in Figs. 4b, 13f). There is a large literature on the applications of stripe patterns in real organisms and chemical models (Ouyang and Swinney 1991; Nagorcka and Mooney 1992; Kondo and Asai 1995) (and this is something that Turing mentioned in his original 1952 paper), as well as a large literature on their stability and other theoretical properties (Ermentrout 1991; Lyons and Harrison 1991; Moyles et al. 2016).

In the case of the planar ellipse, we will consider initial data of the form

59 $$\begin{aligned} \mathbf {u}(0) = \mathbf {u}^* + \cos (2 k \pi \alpha )(1,-1)^T, \end{aligned}$$

where $\mathbf {u}^*$ is again the spatially homogeneous steady state, $\alpha \in [0,1]$ is the radial coordinate on the unit disk, and *k* is a positive integer which determines the number of circular stripes in the target pattern. The vector of opposite signs is used as we will only consider the Schnakenberg kinetics with parameters that give rise to stripe solutions (**S2**), and the activator and inhibitor are always out of phase as is typical for models with cross-kinetics. Depending on the size of the domain, these kinetics and parameter choices lead to a stable pattern very close to the original one, with $k-1$ interior stripes, one stripe along the boundary, and a single central spot completing the target pattern. One can readily generate a bifurcation diagram in radius $r_0$ and *k* and numerically observe stability of different modes, but for brevity we set $k=2$ and $r_0=20$ in the elliptical case, and consider growth up to $r_T=4r_0=80$.

**Fig. 16:**
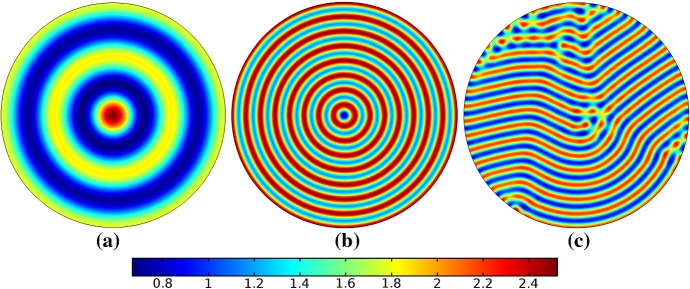
Simulations of Eq. (25) with kinetics given by (44) using parameters S2. We plot the distribution of *u*. **a** is the pattern at time $T^*=6\times 10^4$ on the initial domain, **b** is the final stationary pattern at the time $T=7.2\times 10^5$ in this slow growth case, and **c** is the final pattern for an isotropic growth timescale of $T_g=10^3$ (Color figure online)

We plot the stationary pattern on the initial domain, as well as a simulation using isotropic growth in Fig. 16. We note that simulations using $k=2$ on the static final domain become unstable and evolve into the same pattern shown in Fig. 16b, which corresponds to $k=8$ without a central spot. These patterns are stable for growth timescales larger than $10^4$, but as shown in Fig. 16c, defects emerge and destroy the perfectly symmetric rings if the growth is too rapid. Likely there is an interplay here between numerical discretization errors, errors in the initial data, and symmetry of growing target patterns, but we leave further exploration of these intertwined effects to future work. We also find that staged or anisotropic growth of any form appears to destroy symmetric stripe patterns, in all simulations we have considered.

**Fig. 17:**
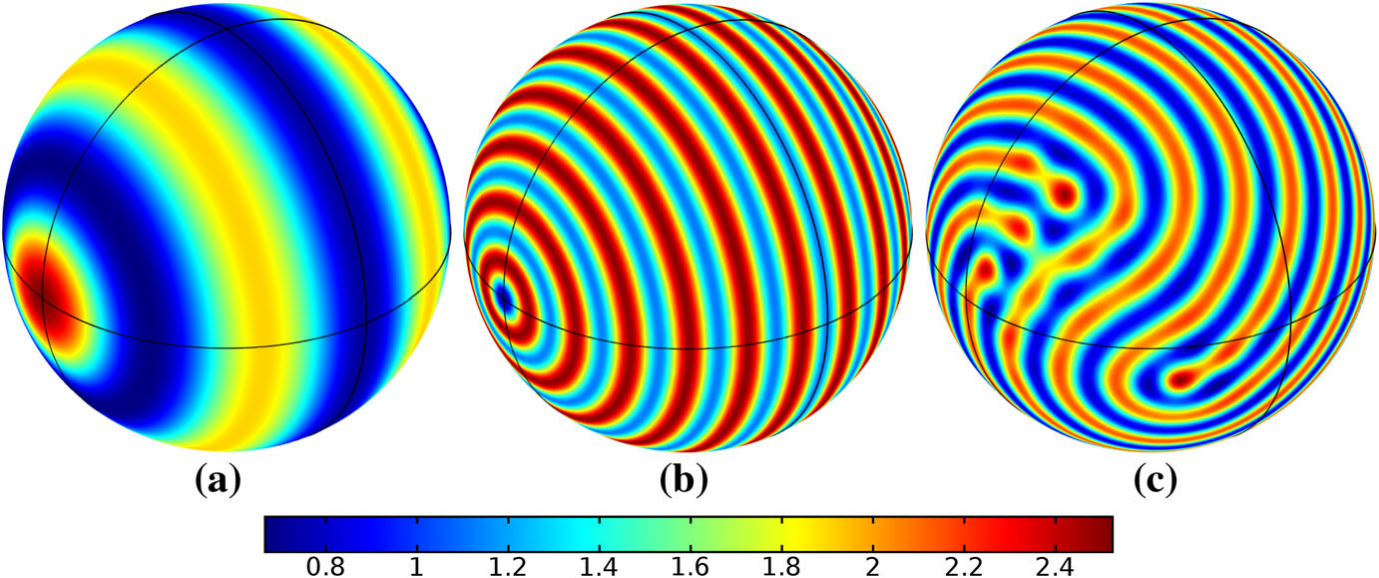
Simulations of Eq. (41) with kinetics given by (44) using parameters S2. We plot the distribution of *u*, **a** is the pattern at time $T^*=6\times 10^4$ on the initial domain, **b** is the final stationary pattern on the static final domain, and **c** is the final pattern for an isotropic growth timescale of $T_g=10T^*=6\times 10^5$ (Color figure online)

Lastly we consider analogous patterns on the ellipsoid. We use a similar form of the initial data as in the case of the ellipse, but project it along one axis, so that the initial pattern develops into targets with surrounding stripes at antipodal points of the *y* axis. In the extrinsic and stationary Cartesian frame, this is given by

60 $$\begin{aligned} \mathbf {u}(0) = \mathbf {u}^* + \cos (2 k \pi x )(1,-1)^T, \end{aligned}$$

where $x \in [-1, 1]$. In Fig. 17, we show the stationary pattern on the initial domain, the final static domain, and the case of slow isotropic growth, as shown in Fig. 16b. We note that the static domain also breaks up into a higher-mode pattern, but growth distorts the pattern by introducing defects throughout the striped patterns. This is likely because isotropic growth of a sphere is not isotropic with respect to the striped pattern, so growth destabilizes these stripes, leading to labyrinthine patterning. This is inherently an effect due to the curvature of the domain not present in planar isotropic growth.

**Fig. 18:**
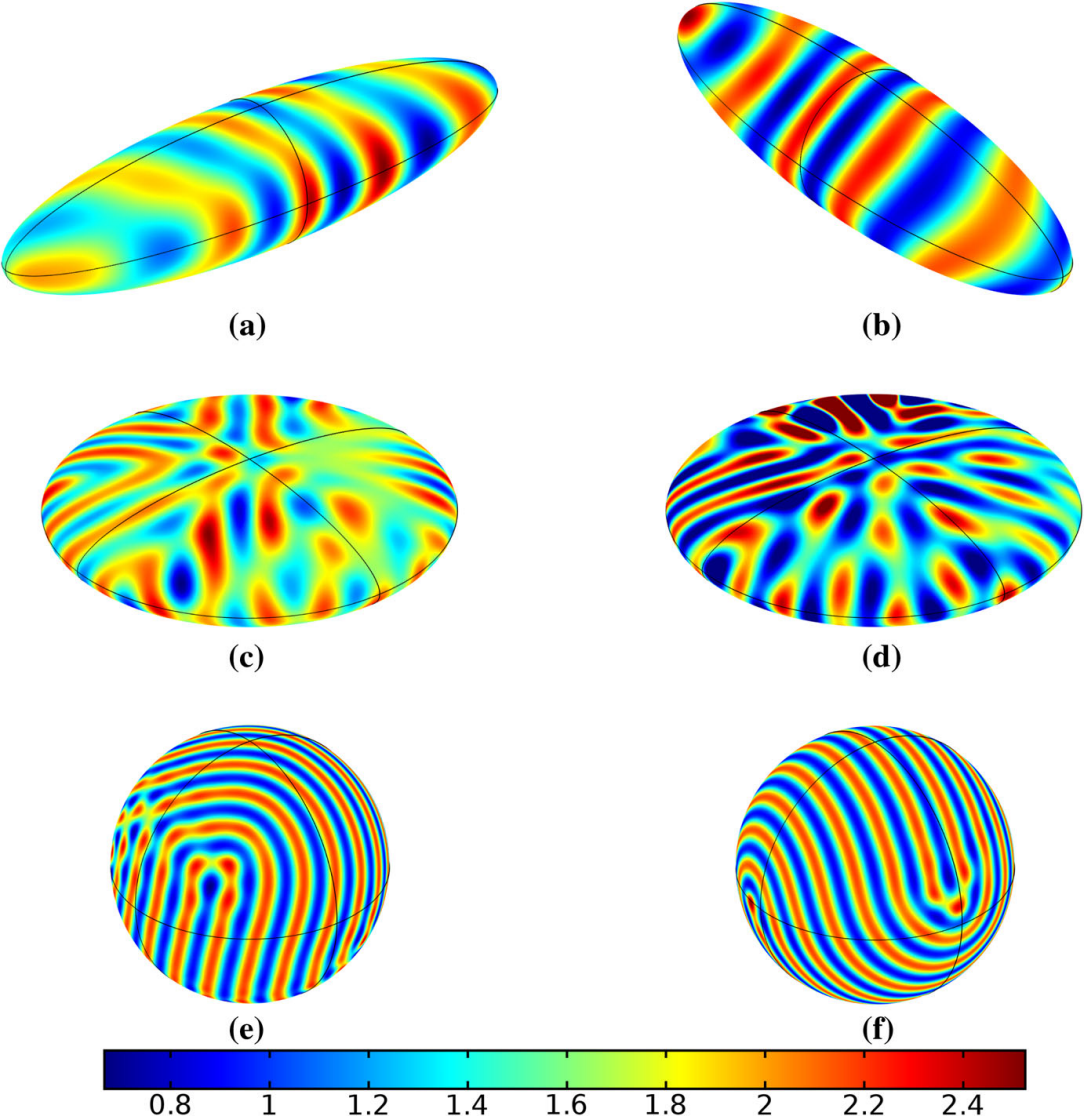
Simulations of Eq. (41) with kinetics given by (44) using parameters S2. We plot the distribution of *u*. In **a**, **c**, **e**, we show staged growth along axes *x*, *y*, and then *z*, whereas in **b**, **d**, and **f** we show staged growth along axes *y*, *x*, and finally *z*. These are at times **a**, **b**$6.6\times 10^5$, **c**, **d**$1.26\times 10^6$, and **e**, **f**$1.92\times 10^6$ (Color figure online)

For staged growth, we show some example simulations in Fig. 18 which all correspond to different stages and directions of anisotropy given the same initial state shown in Fig. 17a. Independent of the initial direction of growth, we see staged growth develop into an approximate target pattern for an elongated ellipsoid, but further growth along other axes leads to more complicated labyrinthine patterning, leading to the final patterns comparable to 17c. Compared with Fig. 16, the target patterns on curved domains appear much less stable to growth, likely due to the influence of curvature.

## Discussion

Extending the work of Plaza et al. (2004), we have generalized reaction–diffusion systems on growing two-dimensional manifolds to include situations where the growth is anisotropic yet dilational in nature. This allowed us to systematically study the evolution of patterns in a variety of growth scenarios, in order to understand how growth affects the robustness of non-equilibrium patterns. We primarily concentrated on patterns which naturally emerged from Turing instabilities, but also gave some examples of stripe patterns, and their sensitivity to growth and curvature. While the restriction to only dilational anisotropy is somewhat restrictive, it allows for an exploration of the role that anisotropy has on the evolution of patterns under growth in a wide variety of growth scenarios.

Our results show several key findings. Firstly, the growth rate and kind of growth (e.g., isotropic or anisotropic) can significantly change the final pattern, even though we can demonstrate that the final pattern is a steady state. This suggests that the high level of multistability in these systems on fixed domains can be heavily influenced by their history due to growth, as suggested by Klika and Gaffney (2017). However, the functional form of growth was never really important; all simulations using different forms of the growth rates were qualitatively the same with linear growth at a suitable rate, as long as the initial and final manifolds were fixed. Secondly, we have used anisotropy in staged growth as compared to both quasi-static growth and isotropic growth to the same final manifold, as a concrete way of demonstrating the importance of both anisotropy and history in determining patterning. A final key finding is that the qualitative nature of patterns heavily depends on both the nonlinear reaction kinetics under consideration and the specific parameter regimes studied. While we only explored three candidate choices of reaction kinetics, we believe these are good exemplars of reaction–diffusion systems sufficient to obtain a qualitative picture of the influence of growth on patterned states.

Spot patterns in Gierer–Meinhardt and Schnakenberg were often not influenced by growth (Figs. 1, 2, 5) and manifold curvature only gave rise to slight local distortions in spots (Fig. 12b–c). The conservation of spots in the Gierer–Meinhardt system is something that has not, to our knowledge, been reported in the literature. Specifically, dropping the constant term in the first of Eq. (47) and letting spot patterns stabilize before growth always led to no new spots forming, independent of the manner of growth. With this feed rate term, however, the behavior of the Gierer–Meinhardt system then became heavily dependent on both the parameters used, as well as the growth rate. ‘Large’ spot solutions behaved as in Schnakenberg, with comparable numbers and distributions of spots in both quasi-static and grown domains. The smaller spot solutions, in Fig. 6, showed a dependence on the growth rate wherein slow enough growth permitted fewer final spots in the final domain. Finally, spot solutions arising from the FitzHugh–Nagumo kinetics exhibited an interesting dependence on both isotropy and curvature (see Figs. 7, 8, 9, 14). Isotropic growth led to labyrinthine-like patterns, whereas staged growth had an obvious effect on the curved regions. For slow growth rates, the total number of such regions was the same between the initial domain and the final one, whereas fast growth could initiate the formation of new regions, akin to spot splitting due to growth. This stark contrast between these reaction kinetics suggests that excitable media might behave quite differently under growth, compared to their dissipative counterparts.

Labyrinthine patterns exhibited a similar richness of behavior. Using Schnakenberg kinetics, stripes could be aligned if the domain was static, but not symmetric, whereas growth would destabilize such alignment (Fig. 3). For some growth rates and kinds of growth, these solutions could spontaneously form target patterns with symmetric stripes (Figs. 4b, 13d). Solutions for FitzHugh–Nagumo kinetics displayed slightly different behaviors, but also showed a strong dependence on anisotropy (Figs. 10, 11, 15). In this case, we did not see the emergence of target patterns or similarly perfectly symmetric structures, but we suspect that for some parameters this might be possible.

Finally, we explored the evolution of striped patterns in Sect. 6. These findings reinforce the key roles of growth rates, anisotropy, and curvature in determining the properties of the final steady-state patterns. It is interesting that in any growing ellipsoid we do not find any stable target or striped pattern that is not a labyrinthine-like pattern. This may be due to the specific parameter choices we have made, and we leave further investigation of stripes and other specific kinds of patterns to future work.

We have demonstrated the complex influence of growth, curvature, and anisotropy on non-uniform patterns arising from Turing instabilities on planar and curved manifolds. There are many possible extensions to our work, including considering manifolds with holes or cusps, or considering bulk-surface reaction–diffusion systems as in Madzvamuse et al. (2015), under the influence of growth. Additionally, further generalizing the reaction–diffusion framework to account for arbitrary non-uniform growth would allow for more accurate modeling of realistic pattern formation in development and elsewhere. Finally, our work also shows behaviors of non-uniform solutions to nonlinear PDE in complicated domains, and the stability and evolution of such solutions are usually confined to illustrative special cases, as in Trinh and Ward (2016). One could pursue similar analyses in the context of anisotropic growth, in order to deepen our understanding of the effects we have demonstrated here.

## References

[CR1] Amar MB, Jia F (2013). Anisotropic growth shapes intestinal tissues during embryogenesis. Proc Natl Acad Sci.

[CR2] Barrass I, Crampin EJ, Maini PK (2006). Mode transitions in a model reaction–diffusion system driven by domain growth and noise. Bull Math Biol.

[CR3] Barreira R, Elliott CM, Madzvamuse A (2011). The surface finite element method for pattern formation on evolving biological surfaces. J Math Biol.

[CR4] Bittig T, Wartlick O, Kicheva A, González-Gaitán M, Jülicher F (2008). Dynamics of anisotropic tissue growth. New J Phys.

[CR5] Borckmans P., Dewel G., De Wit A., Walgraef D. (1995). Turing Bifurcations and Pattern Selection. Chemical Waves and Patterns.

[CR6] Cartwright JH (2002) Labyrinthine Turing pattern formation in the cerebral cortex. arXiv preprint arXiv:nlin/0211001 10.1006/jtbi.2002.3012 12183134

[CR7] Castillo JA, Sánchez-Garduño F, Padilla P (2016). A Turing-Hopf bifurcation scenario for pattern formation on growing domains. Bull Math Biol.

[CR8] Comanici A, Golubitsky M (2008). Patterns on growing square domains via mode interactions. Dyn Syst.

[CR9] Corson F, Hamant O, Bohn S, Traas J, Boudaoud A, Couder Y (2009). Turning a plant tissue into a living cell froth through isotropic growth. Proc Natl Acad Sci.

[CR10] Crampin E, Maini P (2001). Reaction–diffusion models for biological pattern formation. Methods Appl Anal.

[CR11] Crampin EJ, Gaffney EA, Maini PK (1999). Reaction and diffusion on growing domains: scenarios for robust pattern formation. Bull Math Biol.

[CR12] Crampin E, Hackborn W, Maini P (2002). Pattern formation in reaction–diffusion models with nonuniform domain growth. Bull Math Biol.

[CR13] Cross MC, Hohenberg PC (1993). Pattern formation outside of equilibrium. Rev Mod Phys.

[CR14] Dziuk G, Elliott CM (2007). Finite elements on evolving surfaces. IMA J Numer Anal.

[CR15] Dziuk G, Elliott CM (2013). Finite element methods for surface PDEs. Acta Numer.

[CR16] Ermentrout B (1991). Stripes or spots? Nonlinear effects in bifurcation of reaction–diffusion equations on the square. Proc R Soc Lond A.

[CR17] FitzHugh R (1955). Mathematical models of threshold phenomena in the nerve membrane. Bull Math Biol.

[CR18] FitzHugh R (1961). Impulses and physiological states in theoretical models of nerve membrane. Biophys J.

[CR19] Gierer A, Meinhardt H (1972). A theory of biological pattern formation. Kybernetik.

[CR20] Green JB, Sharpe J (2015). Positional information and reaction–diffusion: two big ideas in developmental biology combine. Development.

[CR21] Hetzer G, Madzvamuse A, Shen W (2012). Characterization of Turing diffusion-driven instability on evolving domains. Discrete Contin Dyn Syst Ser A.

[CR22] Hodgkin AL, Huxley AF (1952). A quantitative description of membrane current and its application to conduction and excitation in nerve. J Physiol.

[CR23] Hunding A (1980). Dissipative structures in reaction–diffusion systems: numerical determination of bifurcations in the sphere. J Chem Phys.

[CR24] Iron D, Wei J, Winter M (2004). Stability analysis of Turing patterns generated by the Schnakenberg model. J Math Biol.

[CR25] Jensen O, Pannbacker VO, Dewel G, Borckmans P (1993). Subcritical transitions to Turing structures. Phys Lett A.

[CR26] Keener JP, Sneyd J (1998). Mathematical physiology.

[CR27] Klika V, Gaffney EA (2017). History dependence and the continuum approximation breakdown: the impact of domain growth on turings instability. Proc R Soc A.

[CR28] Kondo S, Asai R (1995). A reaction–diffusion wave on the skin of the marine angelfish pomacanthus. Nature.

[CR29] Krause AL, Burton AM, Fadai NT, Van Gorder RA (2018). Emergent structures in reaction–advection–diffusion systems on a sphere. Phys Rev E.

[CR30] Krause AL, Klika V, Woolley TE, Gaffney EA (2018). Heterogeneity induces spatiotemporal oscillations in reaction–diffusion systems. Phys Rev E.

[CR31] Liu P, Shi J, Wang Y, Feng X (2013). Bifurcation analysis of reaction–diffusion Schnakenberg model. J Math Chem.

[CR32] Lyons M, Harrison L (1991). A class of reaction–diffusion mechanisms which preferentially select striped patterns. Chem Phys Lett.

[CR33] Macdonald CB, Merriman B, Ruuth SJ (2013). Simple computation of reaction–diffusion processes on point clouds. Proc Natl Acad Sci.

[CR34] Madzvamuse A, Barreira R (2014). Exhibiting cross-diffusion-induced patterns for reaction–diffusion systems on evolving domains and surfaces. Phys Rev E.

[CR35] Madzvamuse A, Maini PK (2007). Velocity-induced numerical solutions of reaction–diffusion systems on continuously growing domains. J Comput Phys.

[CR36] Madzvamuse A, Wathen AJ, Maini PK (2003). A moving grid finite element method applied to a model biological pattern generator. J Comput Phys.

[CR37] Madzvamuse A, Gaffney EA, Maini PK (2010). Stability analysis of non-autonomous reaction–diffusion systems: the effects of growing domains. J Math Biol.

[CR38] Madzvamuse A, Chung AH, Venkataraman C (2015). Stability analysis and simulations of coupled bulk-surface reaction–diffusion systems. Proc R Soc A.

[CR39] Maini PK, Woolley TE, Baker RE, Gaffney EA, Lee SS (2012). Turing’s model for biological pattern formation and the robustness problem. Interface Focus.

[CR40] Marcon L, Diego X, Sharpe J, Müller P (2016). High-throughput mathematical analysis identifies Turing networks for patterning with equally diffusing signals. Elife.

[CR41] Menzel A (2005). Modelling of anisotropic growth in biological tissues. Biomech Model Mechanobiol.

[CR42] Moyles I, Tse W, Ward M (2016). Explicitly solvable nonlocal eigenvalue problems and the stability of localized stripes in reaction–diffusion systems. Stud Appl Math.

[CR43] Murray JD (2003). Mathematical biology. II. Spatial models and biomedical applications. Interdisciplinary applied mathematics.

[CR44] Nagorcka B, Mooney J (1992). From stripes to spots: prepatterns which can be produced in the skin by a reaction–diffusion system. Math Med Biol.

[CR45] Nagumo J, Arimoto S, Yoshizawa S (1962). An active pulse transmission line simulating nerve axon. Proc IRE.

[CR46] Núñez-López M, Chacón-Acosta G, Santiago J (2017). Diffusion-driven instability on a curved surface: spherical case revisited. Braz J Phys.

[CR47] Olshanskii MA, Xu X (2017). A trace finite element method for PDEs on evolving surfaces. SIAM J Sci Comput.

[CR48] Ouyang Q, Swinney HL (1991). Transition from a uniform state to hexagonal and striped Turing patterns. Nature.

[CR49] Page KM, Maini PK, Monk NA (2005). Complex pattern formation in reaction–diffusion systems with spatially varying parameters. Phys D Nonlinear Phenom.

[CR50] Peaucelle A, Wightman R, Höfte H (2015). The control of growth symmetry breaking in the Arabidopsis hypocotyl. Curr Biol.

[CR51] Plaza RG, Sanchez-Garduno F, Padilla P, Barrio RA, Maini PK (2004). The effect of growth and curvature on pattern formation. J Dyn Differ Equ.

[CR52] Rossi F, Duteil NP, Yakoby N, Piccoli B (2016) Control of reaction–diffusion equations on time-evolving manifolds. In: 2016 IEEE 55th conference on decision and control (CDC). IEEE, pp 1614–1619 10.1109/CDC.2016.7798496 PMC5634711 29026267

[CR53] Saez A, Ghibaudo M, Buguin A, Silberzan P, Ladoux B (2007). Rigidity-driven growth and migration of epithelial cells on microstructured anisotropic substrates. Proc Natl Acad Sci.

[CR54] Sarfaraz W, Madzvamuse A (2017). Classification of parameter spaces for a reaction–diffusion model on stationary domains. Chaos Solitons Fractals.

[CR55] Satnoianu RA, Menzinger M, Maini PK (2000). Turing instabilities in general systems. J Math Biol.

[CR56] Schnakenberg J (1979). Simple chemical reaction systems with limit cycle behaviour. J Theor Biol.

[CR57] Townsend A, Trefethen LN (2013). An extension of Chebfun to two dimensions. SIAM J Sci Comput.

[CR58] Townsend A, Wilber H, Wright GB (2016). Computing with functions in spherical and polar geometries I. The sphere. SIAM J Sci Comput.

[CR59] Trinh PH, Ward MJ (2016). The dynamics of localized spot patterns for reaction–diffusion systems on the sphere. Nonlinearity.

[CR60] Tse WH, Wei J, Winter M (2010). The Gierer–Meinhardt system on a compact two-dimensional Riemannian manifold: interaction of Gaussian curvature and Green’s function. Journal de Mathématiques Pures et Appliquées.

[CR61] Tuncer N, Madzvamuse A (2017). Projected finite elements for systems of reaction–diffusion equations on closed evolving spheroidal surfaces. Commun Comput Phys.

[CR62] Turing AM (1952). The chemical basis of morphogenesis. Philos Trans R Soc Lond Ser B Biol Sci.

[CR63] Ubeda-Tomás S, Swarup R, Coates J, Swarup K, Laplaze L, Beemster GT, Hedden P, Bhalerao R, Bennett MJ (2008). Root growth in Arabidopsis requires gibberellin/DELLA signalling in the endodermis. Nat Cell Biol.

[CR64] van Saarloos W, van Hecke M, Hohenberg P (1994). Amplitude equations for pattern forming systems. Fundam Probl Stat Mech.

[CR65] Varea C, Aragón J, Barrio R (1997). Confined Turing patterns in growing systems. Phys Rev E.

[CR66] Wei J, Winter M (2013). Mathematical aspects of pattern formation in biological systems.

[CR67] Woolley TE, Baker RE, Maini PK (2017) Turings theory of morphogenesis: where we started, where we are and where we want to go. In: The incomputable. Springer, pp 219–235

